# T-cell responses to ancestral SARS-CoV-2 and Omicron variant among unvaccinated pregnant and postpartum women living with and without HIV in South Africa

**DOI:** 10.1038/s41598-024-70725-8

**Published:** 2024-09-02

**Authors:** William C. McMahon, Gaurav Kwatra, Alane Izu, Stephanie A. Jones, Nkululeko J. Mbele, Nwabisa Jafta, Rushil Lala, Sharon Shalekoff, Caroline T. Tiemessen, Shabir A. Madhi, Marta C. Nunes

**Affiliations:** 1 South African Medical Research Council, Vaccines and Infectious Diseases Analytics Research Unit, Faculty of Health Sciences, University of the Witwatersrand, Johannesburg, South Africa; 2 South African Research Chair Initiative in Vaccine Preventable Diseases, Department of Science and Innovation/National Research Foundation, Faculty of Health Sciences, University of the Witwatersrand, Johannesburg, South Africa; 3 Division of Infectious Diseases, Department of Pediatrics, Cincinnati Children’s Hospital Medical Center and University of Cincinnati, Cincinnati, OH USA; 4 Department of Clinical Microbiology, Christian Medical College, Vellore, India; 5 African Leadership in Vaccinology Expertise, Faculty of Health Sciences, University of the Witwatersrand, Johannesburg, South Africa; 6 A Division of the National Health Laboratory Service, Centre for HIV and STIs, National Institute for Communicable Diseases, Johannesburg, South Africa; 7 Faculty of Health Sciences, University of the Witwatersrand, Johannesburg, South Africa; 8 Center of Excellence in Respiratory Pathogens, Hospices Civils de Lyon, and Centre International de Recherche en Infectiologie, Inserm U1111, CNRS UMR5308, ENS de Lyon, Université Claude Bernard Lyon 1, Lyon, France

**Keywords:** Microbiology, Virology, SARS-CoV-2, Cytokines, Infection, Infectious diseases, Lymphocytes, Immunology, Adaptive immunity, Cellular immunity

## Abstract

SARS-CoV-2 cell-mediated immunity remains understudied during pregnancy in unvaccinated Black African women living with HIV (WLWH) from low- and middle-income countries. We investigated SARS-CoV-2-specific T-cell responses 1 month following infection in 24 HIV-uninfected women and 15 WLWH at any stage during pregnancy or postpartum. The full-length spike (FLS) glycoprotein and nucleocapsid (N) protein of wild-type (WT) SARS-CoV-2, as well as mutated spike protein regions found in the Omicron variant (B.1.1.529) were targeted by flow cytometry. WT-specific CD4^+^ and CD8^+^ T cells elicited similar FLS- and N-specific responses in HIV-uninfected women and WLWH. SARS-CoV-2-specific T-lymphocytes were predominantly TNF-α monofunctional in pregnant and postpartum women living with and without HIV, with fever cells producing either IFN-γ or IL-2. Furthermore, T-cell responses were unaffected by Omicron-specific spike mutations as similar responses between Omicron and the ancestral virus were detected for CD4^+^ and CD8^+^ T cells. Our results collectively demonstrate comparable T-cell responses between WLWH on antiretroviral therapy and HIV-uninfected pregnant and postpartum women who were naïve to Covid-19 vaccination. Additionally, we show that T cells from women infected with the ancestral virus, Beta variant (B.1.351), or Delta variant (B.1.617.2) can cross-recognize Omicron, suggesting an overall preservation of T-cell immunity.

## Introduction

Pregnant women infected with severe acute respiratory syndrome coronavirus 2 (SARS-CoV-2) are at increased risk of developing severe coronavirus disease 2019 (Covid-19) and may experience obstetric complications including preeclampsia^1,2^ and adverse birth outcomes such as preterm birth^2,3^ and stillbirth^4,5^. Similarly, poor pregnancy outcomes have been reported following infections with other respiratory viruses, such as influenza A (H1N1)^6^, severe acute respiratory syndrome coronavirus 1 (SARS-CoV-1)^7^, and Middle East respiratory syndrome coronavirus (MERS-CoV)^8,9^. Physiological and immunological adaptations that occur during pregnancy to allow the mother to tolerate the semi-allogeneic foetus also increase the host’s susceptibility to microbial infections^10,11^. While pregnancy is not considered an immunosuppressive state, pregnant women differ immunologically from non-pregnant women^12,13^. Despite the proven safety and efficacy profiles of Covid-19 vaccines in pregnant women, vaccine hesitancy during pregnancy remains high^13–15^.

SARS-CoV-2 infection during pregnancy among women living with human immunodeficiency virus type 1 (WLWH; HIV-1) remains understudied. HIV infection is characterised by a gradual reduction of absolute CD4^+^ T cells, leading to impaired cellular immunity and increased susceptibility to opportunistic infections^16^. CD4^+^ T cells, together with CD8^+^ T cells and B cells, constitute the three fundamental components of adaptive immunity and are responsible for controlling most viral infections^17^. SARS-CoV-2 infection induces robust cell-mediated CD4^+^ T-cell responses that promote antibody production by B cells, as well as assist CD8^+^ T cells in mediating cytotoxic lysis of SARS-CoV-2 infected cells^17^. Furthermore, SARS-CoV-2 is also capable of infecting monocytes, as well as B cells and CD4^+^ T cells, but not CD8^+^ T cells^18–20^. Since lymphocytes only express low levels of angiotensin-converting enzyme 2 (ACE2), it has been shown that SARS-CoV-2 uses alternative mechanisms or auxiliary molecules located on lymphocyte plasma membranes to gain cellular entry^20,21^. CD4, a membrane glycoprotein mainly expressed on T helper cells and known co-receptor of HIV, interacts with the spike glycoprotein of SARS-CoV-2 to promote cellular entry, causing lymphocytopenia and impaired inflammatory responses^20^.

Although the incidence of SARS-CoV-2 infection is similar between people living with HIV (PWH) and the general population, PWH who are immunosuppressed or not receiving antiretroviral therapy (ART) are at increased risk of developing severe disease^22^. Given the high HIV burden in sub-Saharan African countries^23^, as well as the risks of SARS-CoV-2 infection during pregnancy, it is critical to investigate immune responses to SARS-CoV-2 infection and associated variants in WLWH. Most studies have only compared humoral responses between pregnant and non-pregnant women following SARS-CoV-2 infection or Covid-19 vaccination^13,15,24–26^. However, there is a paucity of information on SARS-CoV-2 cell-mediated immune responses in PWH from low- and middle-income settings^12^.

Here, we report on SARS-CoV-2-specific T-cell responses to ancestral Wuhan wild-type (WT) SARS-CoV-2 and the Omicron variant (B.1.1.529) among unvaccinated pregnant and postpartum women living with and without HIV in South Africa, one month following infection with either WT SARS-CoV-2, the Beta variant (B.1.351), or the Delta variant (B.1.617.2).

## Results

### Characteristics of enrolled participants

A total of 24 HIV-uninfected women (*n* = 12 pregnant, *n* = 12 postpartum) and 15 WLWH (*n* = 5 pregnant, *n* = 5 postpartum, *n* = 5 not pregnant) were recruited with a median age of 30 years (IQR 22–33) and 34 years (IQR 29–40; *p* = 0.035), respectively. Most of the participants (56.4%, 22/39) had been infected with the Beta variant (B.1.351) during the second Covid-19 wave, followed by 25.6% (10/39) with the ancestral strain during the first wave, and 18% (7/39) with the Delta variant (B.1.617.2) during the third wave. PBMCs were isolated earlier from WLWH (30 days, IQR 22–37) compared with HIV-uninfected women (37 days, IQR 33–44; *p* = 0.006), either during pregnancy or during the postpartum period, after confirmed SARS-CoV-2 infection. The median gestational age at the time of positive SARS-CoV-2 NAAT was 38 weeks (IQR 34–39) among WLWH and 38 weeks (IQR 33–39; *p* = 0.704) for HIV-uninfected women. All participants were otherwise healthy, with only 13% (3/24) of the HIV-uninfected women having hypertension and 7% (1/15) of WLWH having anaemia. Covid-19 related symptoms at recruitment were uncommon, with 15.4% (6/39) of participants presenting with coughing, and 2.6% (1/39) with either shortness of breath, sore throat, or diminished taste.

Among WLWH, the median CD4^+^ T-cell count was 392 cells/µL (IQR 239–725) and was similar between the women who were pregnant, postpartum, and not pregnant. 67% (10/15) of WLWH had undetectable HIV-1 viral loads of < 50 RNA copies/mL, whereas 33% (5/15) had HIV-1 viral loads between 50 and 500 RNA copies/mL. All WLWH received fixed-dose combination ART, with 80% (12/15) receiving one non-nucleoside reverse transcriptase inhibitor (NNRTI; Efavirenz) with two nucleoside or nucleotide reverse transcriptase inhibitors (NRTIs; Tenofovir and Lamivudine/Emtricitabine), and 20% (3/15) receiving one integrase strand transfer inhibitor (INSTI; Dolutegravir) with two NRTIs (Tenofovir and Lamivudine). The participants’ characteristics are summarized in Table 1.

**Table 1:** Characteristics of SARS-CoV-2 infected women, stratified by HIV status.

Variable	HIV-uninfected women				Women living with HIV					Overall *p*-value^f^
	Pregnant	Postpartum	*p*-value	Total	Pregnant	Postpartum	Not pregnant	*p*-value	Total	
Number tested	*n* = 12	*n* = 12	–	*N* = 24	*n* = 5	*n* = 5	*n* = 5	–	*N* = 15	–
Age (years), median (IQR)	30 (23–33)	28 (22–36)	0.664	30 (22–33)	40 (33–43)	30 (24–31)	40 (35–40)	0.118	34 (29–40)	0.035
Covid-19 wave (variant) recruitment period, No. (%)^a^										
1st (WT)	4 (33)	3 (25)	–	7 (29)	1 (20)	2 (40)	0 (0)	–	3 (20)	–
2nd (Beta)	6 (50)	7 (58)	–	13 (54)	4 (80)	1 (20)	4 (80)	–	9 (60)	–
3rd (Delta)	2 (17)	2 (17)	–	4 (17)	0 (0)	2 (40)	1 (20)	–	3 (20)	–
SARS-CoV-2 N-gene NAAT CT-value, median (IQR)^b^	37 (33–37)	34 (30–35)	0.317	35 (32–37)	28 (26–32)	36 (33–38)	33 (33–34)	0.195	33 (29–34)	0.443
PBMC isolation post positive SARS-CoV-2 NAAT (days), median (IQR)	41 (36–45)	34 (28–41)	0.056	37 (33–44)	25 (18–33)	30 (23–39)	31 (28–32)	0.623	30 (22–37)	0.006
Gestational age (weeks) at positive SARS-CoV-2 NAAT, median (IQR)^c^	34 (26–39)	38 (36–40)	0.114	38 (33–39)	39 (30–39)	36 (34–40)	N/A	0.806	38 (34–39)	0.704
Comorbidities, No. (%)										
Hypertension	1 (8)	2 (17)	–	3 (13)	0 (0)	0 (0)	0 (0)	–	0 (0)	–
Anaemia	0 (0)	0 (0)	–	0 (0)	1 (20)	0 (0)	0 (0)	–	1 (7)	–
Covid-19-related symptoms at recruitment, No. (%)										
Cough	2 (27)	2 (27)	–	4 (17)	0 (0)	1 (20)	1 (20)	–	2 (13)	–
Shortness of breath	0 (0)	1 (8)	–	1 (4)	0 (0)	0 (0)	0 (0)	–	0 (0)	–
Sore throat	1 (8)	0 (0)	–	1 (4)	0 (0)	0 (0)	0 (0)	–	0 (0)	–
Diminished taste	1 (8)	0 (0)	–	1 (4)	0 (0)	0 (0)	0 (0)	–	0 (0)	–
Percentage T cells, median (IQR)^d^										
CD4^+^ T cells	57.7 (46.9–62.6)	56.6 (42.8–60.9)	0.453	56.8 (45.6–62.6)	38.4 (35.8–46.0)	41.2 (17.2–48.5)	44.9 (38.1–45.9)	0.961	41.22 (35.5–48.5)	0.005
CD8^+^ T cells	33.9 (29.3–39.1)	31.8 (25.9–40.5)	0.603	32.3 (27.3–39.7)	49.7 (49.1–51.1)	51.3 (39.8–66.0)	48.2 (46.1–54.7)	0.914	49.7 (41.5–56.0)	< 0.001
CD4^+^ T-cell count (cells/µL), median (IQR)	–	–	–	–	347 (241–570)	390 (239–392)	644 (551–773)	0.572	392 (239–725)	–
CD4^+^ T-cell count ranges, No. (%)										
< 200 cells/µL	–	–	–	–	1 (20)	0 (0)	1 (20)	–	2 (13)	–
≥ 200–750 cells/µL	–	–	–	–	4 (80)	4 (80)	2 (40)	–	10 (67)	–
≥ 750 cells/µL	–	–	–	–	0 (0)	1 (20)	2 (40)	–	3 (20)	–
HIV-1 viral loads, No. (%)^e^										
< 50 RNA copies/mL	N/A	N/A	N/A	–	3 (60)	3 (60)	4 (80)	–	10 (67)	–
≥ 50–500 RNA copies/mL	N/A	N/A	N/A		2 (40)	2 (40)	1 (20)	–	5 (33)	–
ART regimens, No. (%)										
NNRTI + 2 NRTIs	N/A	N/A	N/A	–	5 (100)	3 (60)	4 (80)	–	12 (80)	–
INSTI + 2 NRTIs	N/A	N/A	N/A	–	0 (0)	2 (40)	1 (20)	–	3 (20)	–

Denotations: *ART* antiretroviral treatment, *CT *cycle threshold value, *HIV-1 *human immunodeficiency virus type 1, *INSTI *integrase strand transfer inhibitor, *IQR *interquartile range, *N/A* not applicable, *NAAT* nucleic acid amplification test, *N-gene* nucleocapsid protein gene, *NNRTI* non-nucleoside reverse transcriptase inhibitor, *NRTI *nucleoside or nucleotide reverse transcriptase inhibitor, *PBMC *peripheral blood mononuclear cells, *RNA *ribonucleic acid, *SARS-CoV-2 *severe acute respiratory syndrome coronavirus 2, *WT* wild-type (ancestral) SARS-CoV-2.

^ a^Recruitment periods during three Covid-19 waves in South Africa, individually driven by WT SARS-CoV-2, the Beta variant (B.1.351), and the Delta variant (B.1.617.2).

^b^The cycle threshold (CT) values for the SARS-CoV-2 N-gene.

^c^Gestational age at recruitment with positive SARS-CoV-2 NAATs.

^d^The percentage CD4^+^ and CD8^+^ T cells as determined by flow cytometry staining after acquiring 100,000 live CD3^+^ cells.

^e^HIV-1 viral loads as determined by the Cobas^®^ 6800/8800 HIV-1 test (Roche Diagnostics International Ltd, Rotkreuz, Switzerland) with a lower limit of detection of 50 RNA copies/mL and linear range of 50–10^7^ RNA copies/mL.

^f^Statistical analyses using the Mann-Whitney U test between two groups and the Kruskal–Wallis H test between three groups. The overall *p*-value represents direct comparisons between HIV-uninfected women and women living with HIV.

### CD4^+^/CD8^+^ T-cell ratio and PD-1 (CD279) expression

**Fig. 1:**
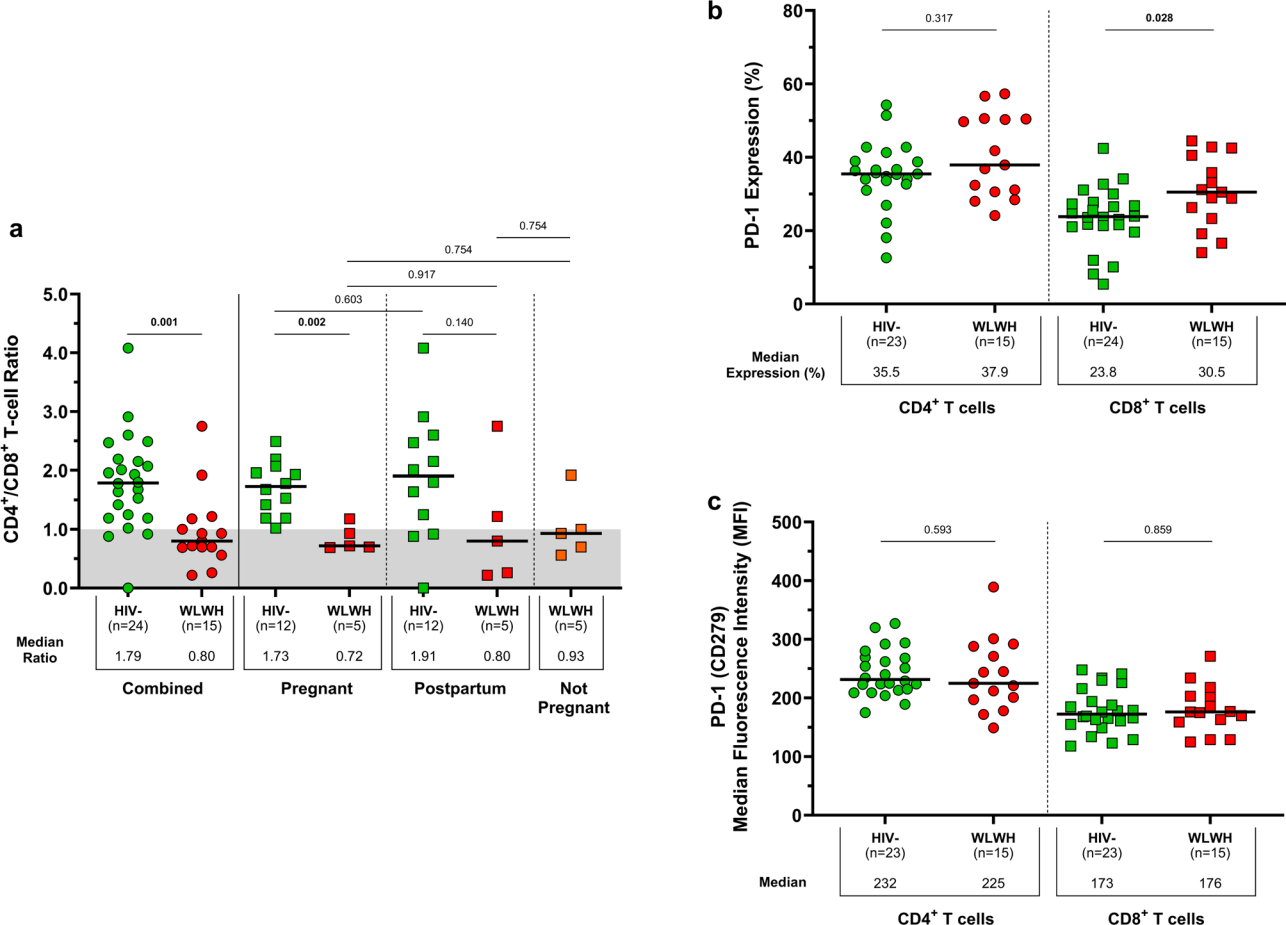
CD4^+^/CD8^+^ T-cell ratios and PD-1 expression in women living with and without HIV. (**a**) Combined CD4^+^/CD8^+^ T-cell ratios in WLWH (*n* = 15) and HIV-uninfected (*n* = 24) women, with individual stratification of CD4^+^/CD8^+^ T-cell ratios in pregnant (*n* = 17), postpartum (*n* = 17), and non-pregnant (*n* = 5) women. Ratios below 1.0 are shaded in grey. (**b**) PD-1 expressing CD4^+^ and CD8^+^ T cells in WLWH (*n* = 15) and HIV-uninfected (*n* = 23) women. (**c**) Median fluorescence intensities of PD-1 on CD4^+^ and CD8^+^ T cells. Each participant is represented by an individual point, and medians in each group are indicated by horizontal lines. The Mann–Whitney U-test and Wilcoxon signed-rank test were used for comparing unpaired (HIV-uninfected vs. WLWH) and paired (HIV-uninfected and WLWH: CD4^+^ T cells vs. CD8^+^ T cells) medians, respectively. Significant *p*-values are denoted in bold. Denotations: *HIV−* HIV-uninfected women, *MFI* median fluorescence intensity, *WLWH* women living with HIV.

The CD4^+^/CD8^+^ T-cell ratio was compared between participants living with and without HIV by quantifying the proportions of live CD3^+^ T cells expressing CD4 and CD8 (Fig. 1a; Table 1). The overall CD4^+^/CD8^+^ T-cell ratio among WLWH was lower compared with HIV-uninfected women (0.80 vs. 1.79; *p* = 0.001). This can be attributed to a greater proportion of CD3^+^ T cells with a CD4^+^ phenotype in HIV-uninfected women compared with WLWH (56.8% vs. 42.1%; *p* = 0.005), and a greater proportion of CD3^+^ T cells with a CD8^+^ phenotype in WLWH compared with HIV-uninfected women (49.7% vs. 32.3%; *p* < 0.001). Among WLWH, the CD4^+^/CD8^+^ T-cell ratio was similar between pregnant women and non-pregnant controls (0.72 vs. 0.93; *p* = 0.754), as well as between postpartum women and non-pregnant controls (0.80 vs. 0.93; *p* = 0.754). HIV-uninfected pregnant women had a higher CD4^+^/CD8^+^ T-cell ratio compared with pregnant WLWH (1.73 vs. 0.72; *p* = 0.002).

Acute and chronic infection is associated with increased expression of programmed cell death protein 1 (PD-1 or CD279). The proportions of CD4^+^ T cells expressing PD-1 were similar between participants living with and without HIV (37.9% vs. 35.5%; *p* = 0.317; Fig. 1b), while proportions of CD8^+^ T cells expressing PD-1 were significantly expanded in WLWH (30.5%) compared with HIV-uninfected women (23.8%; *p* = 0.028). We observed higher frequencies of PD-1-expressing CD4^+^ T cells than PD-1-expressing CD8^+^ T cells (HIV-uninfected: 35.5% vs. 23.8%; *p* < 0.001; WLWH: 37.9% vs. 30.5%; *p* = 0.017), however, these results do not exclusively represent SARS-CoV-2 antigen-activated T cells. T follicular helper cells, a specialised CD4^+^ T-cell subset, naturally express high levels of PD-1 which could have contributed to the increased proportion of PD-1-expressing CD4^+^ T cells. The abundance of PD-1 on CD4^+^ T cells was comparable between HIV-uninfected women and WLWH (MFIs: 232 vs. 225; *p* = 0.593), and was similarly shown for CD8^+^ T cells (MFIs: 173 vs. 176; *p* = 0.859; Fig. 1c).

### T-cell responses to wild-type SARS-CoV-2

**Fig. 2:**
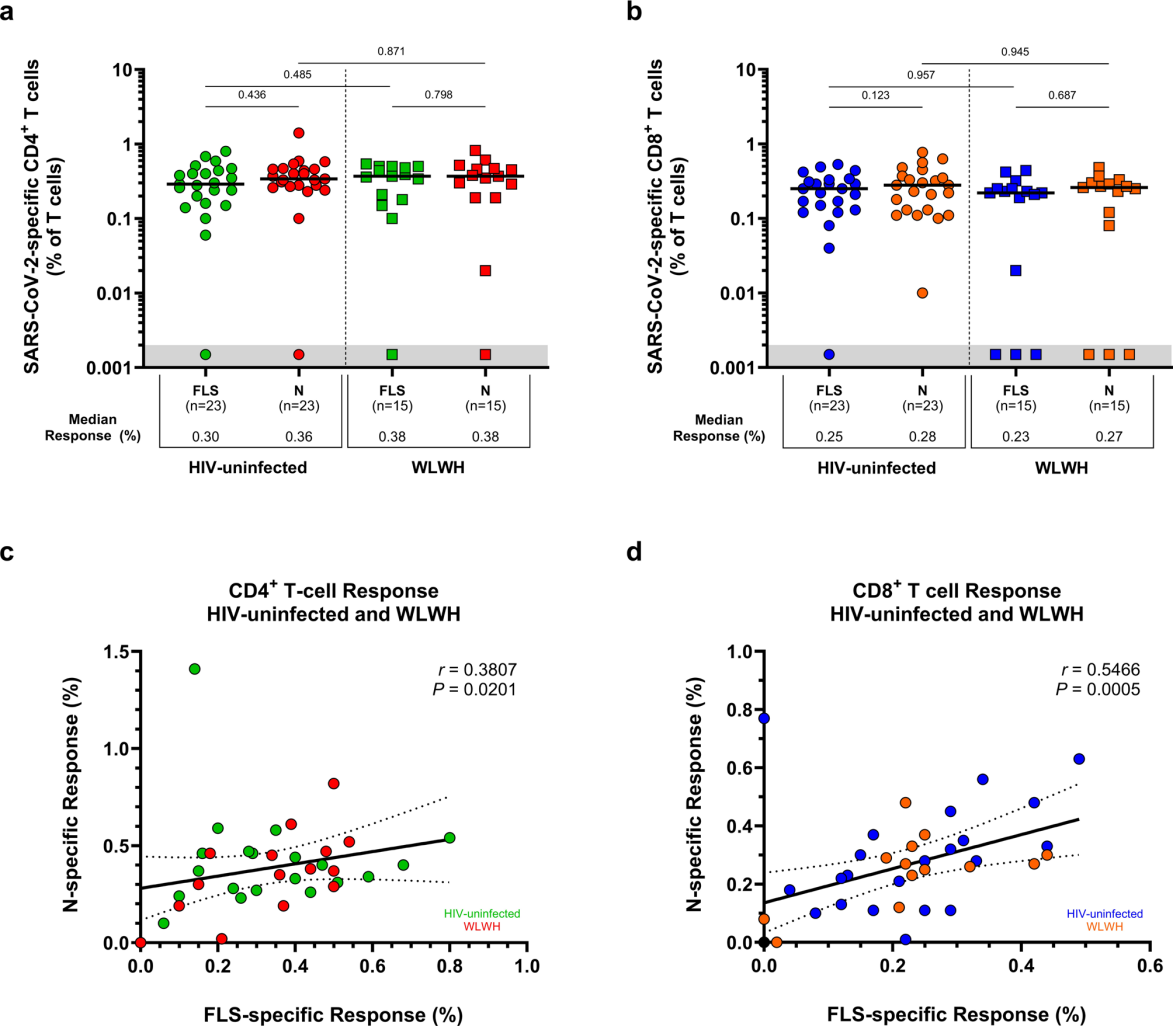
T-cell responses to the FLS and N proteins of WT SARS-CoV-2. The proportion of T cells producing IL-2, IFN-γ, or TNF-α in response to the FLS and N proteins of WT SARS-CoV-2. SARS-CoV-2-specific (**a**) CD4^+^ T-cell responses and (**b**) CD8^+^ T-cell responses. Each participant is represented by an individual point, and medians in each group are indicated by horizontal lines. Statistical analyses were performed using the Mann–Whitney U-test and Wilcoxon signed-rank test for T-cell response comparisons between unpaired (HIV-uninfected vs. WLWH) and paired (HIV-uninfected and WLWH: FLS vs. N) responders, respectively. Correlations between FLS- and N-specific (**c**) CD4^+^ T-cell responses and (**d**) CD8^+^ T-cell responses. Spearman’s rank correlation coefficient (two-sided) was used to determine the *P* and *r* (correlation coefficient) values. Linear regression lines are indicated with 95% confidence interval lines. Denotations: *FLS* full-length spike glycoprotein, *HIV−* HIV-uninfected women, *IFN-γ* interferon gamma, *IL-2* interleukin 2, *N* nucleocapsid protein, *TNF-α* tumor necrosis factor alpha, *WLWH* women living with HIV, *WT* wild-type (ancestral) SARS-CoV-2.

The overall SARS-CoV-2-specific T-cell response was characterised by the ability of T-lymphocytes to produce IL-2, IFN-γ, or TNF-α following PBMC stimulation with peptide antigens covering the FLS and N proteins of WT SARS-CoV-2. CD4^+^ T cells elicited similar FLS- and N-specific responses in HIV-uninfected women (0.30% FLS vs. 0.36% N; *p* = 0.436) and WLWH (0.38% FLS vs. 0.38% N; *p* = 0.798; Fig. 2a). Similar findings were detected for CD8^+^ T cells among HIV-uninfected women (0.25% FLS vs. 0.28% N; *p* = 0.123) and WLWH (0.23% FLS vs. 0.27% N; *p* = 0.687; Fig. 2b). HIV status was not associated (*p* > 0.05) with a difference in the proportion of CD4^+^ and CD8^+^ T cells eliciting a response to the FLS or N peptides of WT SARS-CoV-2, despite a lower CD4^+^/CD8^+^ T-cell ratio in WLWH.

FLS- and N-specific responses elicited by the CD4^+^ T-cell subset correlated modestly with each other, irrespective of HIV status (Spearman’s rank correlation coefficient, *r* = 0.381; *p* = 0.020; Fig. 2c). A stronger association was found for the CD8^+^ T-cell subset (*r* = 0.547; *p* = 0.0005; Fig. 2d). Overall, FLS- and N-specific CD8^+^ T-cell responses were lower compared with CD4^+^ T-cell responses in WLWH and HIV-uninfected women (*p* < 0.05; Supplementary Fig. S2). Additional comparisons of SARS-CoV-2-specific T-lymphocyte responses between pregnant and postpartum women did not yield any differences (Supplementary Fig. S3). Together, these results suggest similar magnitudes of overall SARS-CoV-2-specific T-cell responses directed at the FLS and N proteins of WT SARS-CoV-2 among pregnant and postpartum women living with and without HIV.

### Polyfunctionality profiles of SARS-CoV-2-specific T cells

**Fig. 3:**
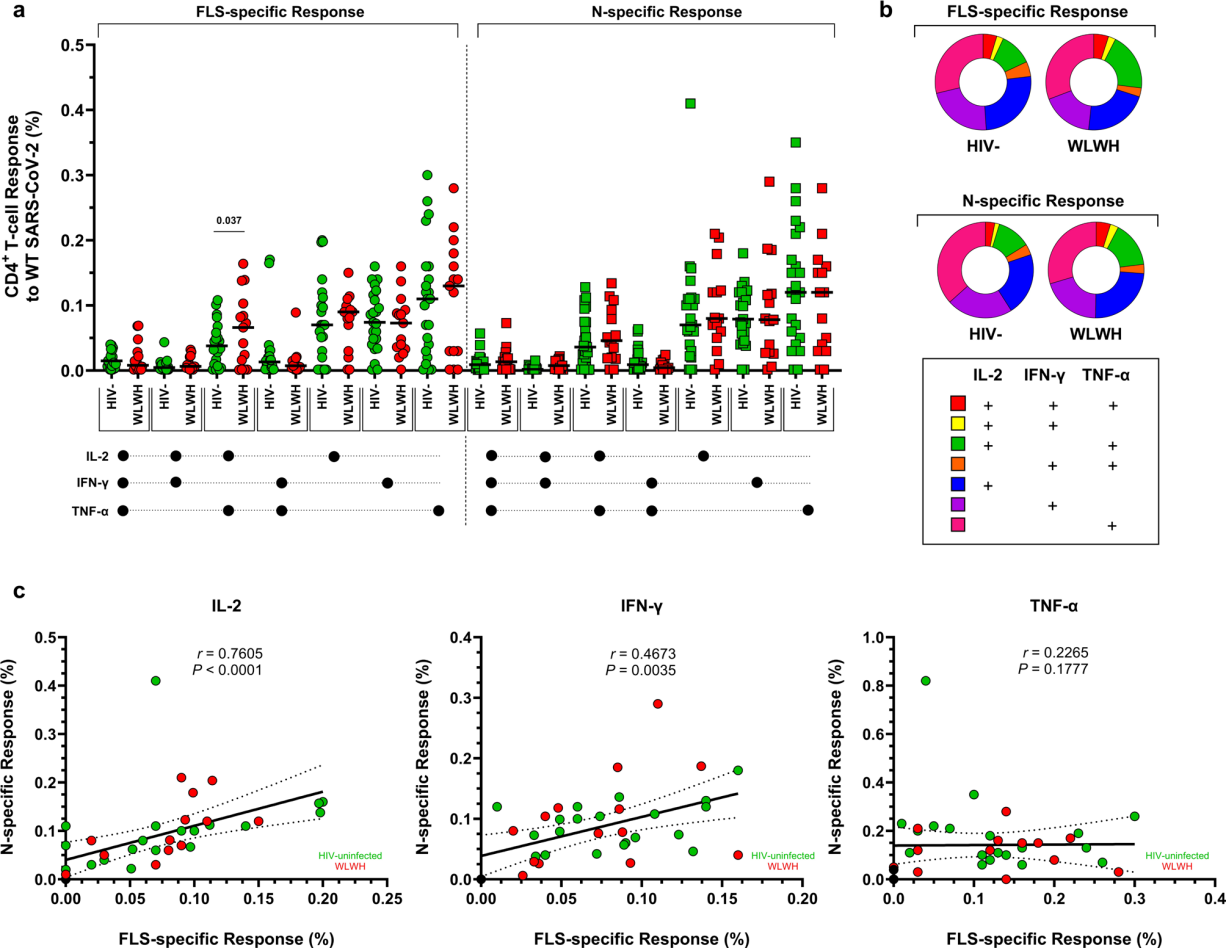
Polyfunctionality of CD4^+^ T cells to the FLS and N proteins of WT SARS-CoV-2. (**a**) Comparisons of the polyfunctional profiles of CD4^+^ T cells to the FLS and N proteins of WT SARS-CoV-2 in HIV-uninfected women and WLWH. Each participant is represented by an individual point, and medians in each group are indicated by horizontal lines. (**b**) Each response pattern is colour-coded, and median response data are summarized in pie charts. The Mann–Whitney U-test was used for comparing unpaired CD4^+^ T-cell responses of positive responders between HIV-uninfected women and WLWH. Only significant response differences are indicated (*p* < 0.05). (**c**) Correlations between FLS- and N-specific CD4^+^ T-cell responses according to IL-2, IFN-γ, and TNF-α production. Spearman’s rank correlation coefficient (two-sided) was used to determine the *P* and *r* (correlation coefficient) values. Linear regression lines are indicated with 95% confidence interval lines. Denotations: *HIV−* HIV-uninfected women, *IFN-γ* interferon gamma, *IL-2* interleukin 2, *N* nucleocapsid protein, *TNF-α* tumor necrosis factor alpha, *WLWH* women living with HIV, *WT* wild-type (ancestral) SARS-CoV-2.

**Fig. 4:**
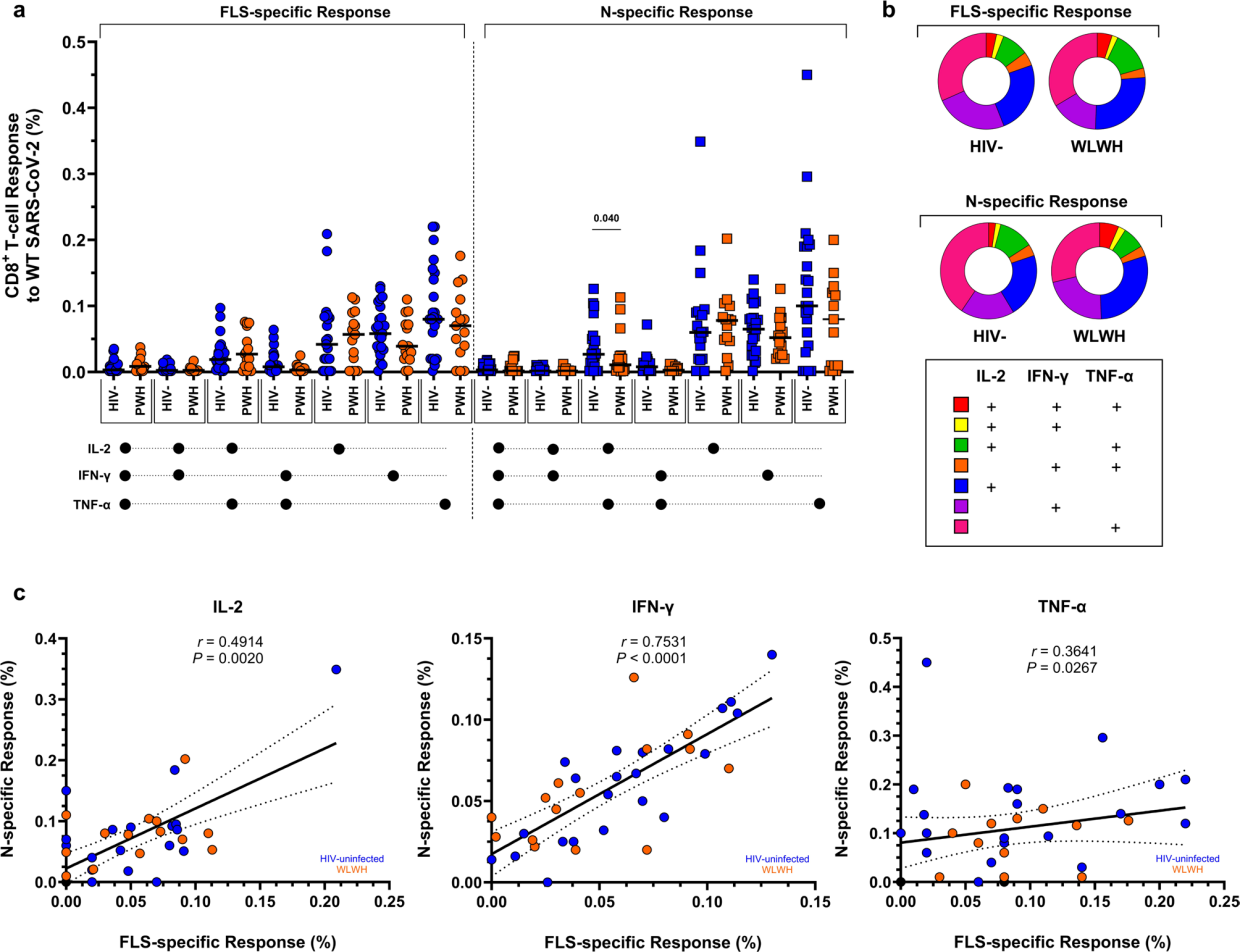
Polyfunctionality of CD8^+^ T cells to the FLS and N proteins of WT SARS-CoV-2. (**a**) Comparisons of the polyfunctional profiles of CD8^+^ T cells to the FLS and N proteins of WT SARS-CoV-2 in HIV-uninfected women and WLWH. Each participant is represented by an individual point, and medians in each group are indicated by horizontal lines. (**b**) Each response pattern is colour-coded, and median response data are summarized in pie charts. The Mann–Whitney U-test was used for comparing unpaired CD8^+^ T-cell responses of positive responders between HIV-uninfected women and WLWH. Only significant response differences are indicated (*p* < 0.05). (**c**) Correlations between FLS- and N-specific CD8^+^ T-cell responses according to IL-2, IFN-γ, and TNF-α production. Spearman’s rank correlation coefficient (two-sided) was used to determine the *P* and *r* (correlation coefficient) values. Linear regression lines are indicated with 95% confidence interval lines. Denotations: *HIV−* HIV-uninfected women, *IFN-γ* interferon gamma, *IL-2* interleukin 2, *N* nucleocapsid protein, *TNF-α* tumor necrosis factor alpha, *WLWH* women living with HIV, *WT* wild-type (ancestral) SARS-CoV-2.

Similar FLS- and N-specific CD4^+^ and CD8^+^ T-cell cytokine response profiles were observed between WLWH and HIV-uninfected women (Figs. 3, 4). SARS-CoV-2-specific T-lymphocytes were mostly TNF-α monofunctional, with fewer cells producing either IFN-γ or IL-2. The highest proportion of T-lymphocytes producing at least two cytokines was seen in the co-production of IL-2 and TNF-α. WLWH had more bifunctional FLS-specific CD4^+^ T cells producing IL-2 and TNF-α compared with HIV-uninfected women (0.082% vs. 0.043%; *p* = 0.037; Fig. 3a,b). Since T-lymphocytes appeared to be predominantly monofunctional, the relationship between the FLS- and N-specific responses to WT SARS-CoV-2 was investigated in single-cytokine-producing cells. CD4^+^ T cells exhibited the strongest correlation in FLS- and N-specific responses to IL-2 production (*r* = 0.761; *p* < 0.0001), followed by IFN-γ (*r* = 0.467; *p* = 0.004; Fig. 3c). Conversely, the strongest correlation between FLS- and N-specific responses in CD8^+^ T cells was found with IFN-γ production (*r* = 0.753; *p* < 0.0001; Fig. 4a,b), followed by IL-2 (*r* = 0.491; *p* = 0.002; Fig. 4c). Despite TNF-α being the most abundant cytokine produced, the associations were absent or less evident with CD4^+^ T cells (*r* = 0.227; *p* = 0.178; Fig. 3c) and CD8^+^ T cells (*r* = 0.364; *p* = 0.027; Fig. 4c), respectively. Comparisons of FLS- and N-specific polyfunctionality profiles were similar between pregnant and postpartum women living with and without HIV (Supplementary Figs. S4–S7). Furthermore, the median fluorescent intensities of IFN-γ, IL-2, and TNF-α on FLS- and N-specific CD4^+^ and CD8^+^ T cells were comparable among women living with and without HIV (Supplementary Fig. S8).

### T-cell cross-reactivity to Omicron

**Fig. 5:**
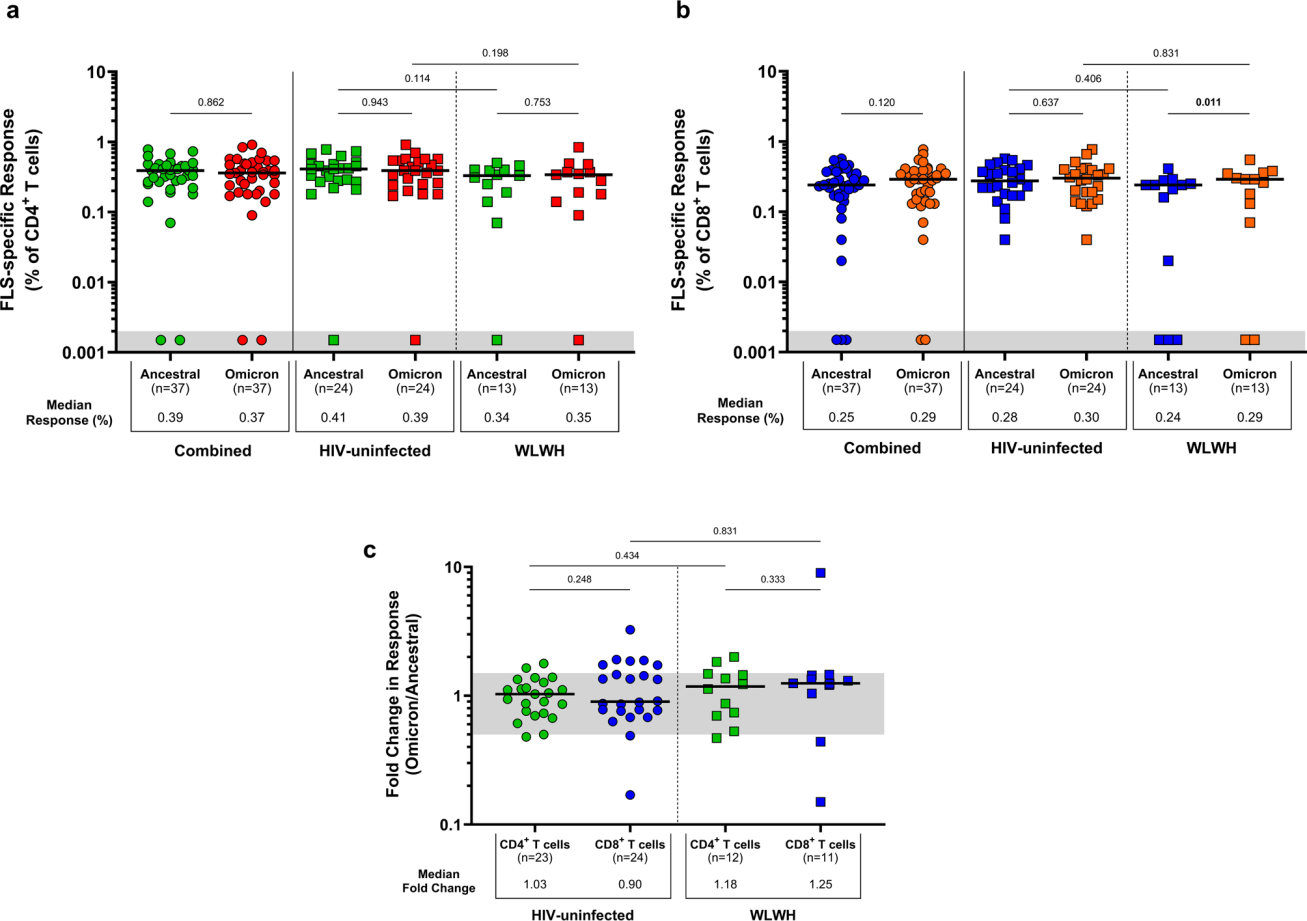
T-cell responses to the spike protein of WT SARS-CoV-2 and the Omicron variant in women living with and without HIV. The proportion of spike-specific T cells producing IL-2, IFN-γ, or TNF-α in response to peptide pools representing partial ancestral and Omicron spike proteins. SARS-CoV-2 specific (**a**) CD4^+^ T-cell responses and (**b**) CD8^+^ T-cell responses. Each participant is represented by an individual point, and medians in each group are indicated by horizontal lines. (**c**) The fold change in spike-specific CD4^+^ and CD8^+^ T-cell responses. Ratios equal to 1.0 indicate equal responses to WT SARS-CoV-2 and Omicron. Ratios below 1.0 indicate greater responses to WT SARS-CoV-2 compared to Omicron. Ratios above 1.0 indicate greater responses to Omicron compared to WT SARS-CoV-2. The threshold area between 0.5 and 1.0 is indicated in grey, representing similar responses to WT SARS-CoV-2 and Omicron. Statistical analyses were performed using the Mann–Whitney U-test and Wilcoxon signed-rank test for comparisons between unpaired (HIV-uninfected vs. WLWH) and paired (HIV-uninfected and WLWH: ancestral vs. Omicron) T-cell responses and fold changes, respectively. Significant *p*-values are indicated in bold (*p* < 0.05). Denotations: *FLS* full-length spike glycoprotein, *HIV-* HIV-uninfected women, *WLWH* women living with HIV, *WT* wild-type (ancestral) SARS-CoV-2.

None of the participants were infected with the Omicron (B.1.1.529) variant, as they were all enrolled during earlier Covid-19 waves before this variant emerged. The ability of participants’ T-lymphocytes to cross-recognise Omicron spike-specific mutations and elicit a detectable response was investigated. The total T-cell response magnitude was characterised by cells producing IL-2, IFN-γ, or TNF-α, rather than solely focusing on single-cytokine-producing cells. Overall, similar CD4^+^ T-cell responses were observed to Omicron peptides compared with WT SARS-CoV-2 peptides (0.37% vs. 0.39%; *p* = 0.862; Fig. 5a), which were similarly shown for CD8^+^ T-cell responses (0.29% vs. 0.25%; *p* = 0.120; Fig. 5b). CD4^+^ T-cell responses to Omicron and ancestral peptides were similar between HIV-uninfected women (0.39% vs. 0.41%; *p* = 0.943) and in WLWH (0.35% vs. 0.34%; *p* = 0.753). Conversely, CD8^+^ T cells in WLWH showed a greater response to Omicron peptides compared with ancestral peptides (0.29% vs. 0.24%; *p* = 0.011). No differences were observed in the median fluorescent intensities of IFN-γ, IL-2, and TNF-α on ancestral- and Omicron-specific CD4^+^ and CD8^+^ T cells among women living with and without HIV (Supplementary Fig. S9).

To characterise spike-specific CD4^+^ and CD8^+^ T-cell responses to Omicron in relation to the ancestral virus, the fold change in these responses was investigated (Fig. 5c). Overall, CD4^+^ and CD8^+^ T-cell responses showed median fold changes above 1.0 in women living with and without HIV, indicating a slightly greater proportion of T cells reacting to Omicron compared with WT SARS-CoV-2. This can be attributed to most participants (74.4%, 29/39) being infected with the Beta (B.1.351) and Delta (B.1.617.2) variants that share some spike-specific mutations with Omicron (B.1.1.529). No differences in the fold change between CD4^+^ and CD8^+^ T-cell responses were detected in women living with and without HIV (*p* > 0.05). Even though 12.8% (5/39) of participants did not elicit a detectable CD4^+^ or CD8^+^ T-cell response to Omicron or WT SARS-CoV-2, these results collectively indicate that T cells from convalescent pregnant and postpartum women living with and without HIV can cross-recognise Omicron.

## Discussion

We showed that T-cell responses to the FLS and N structural proteins of WT SARS-CoV-2 one month following confirmed infection were similar in pregnant and postpartum Black African women living with and without HIV who were naïve to Covid-19 vaccines. Virally suppressed WLWH on ART exhibited comparable polyfunctionality profiles compared to HIV-uninfected women, despite having a lower CD4^+^/CD8^+^ T-cell ratio. Although all women were infected with the ancestral virus, Beta variant (B.1.351), or Delta variant (B.1.617.2) at the time of enrolment, cross-reactive T-cell responses were elicited to Omicron (B.1.1.529) and the ancestral virus to a similar degree, irrespective of HIV status.

The CD4^+^/CD8^+^ T-cell ratio is a biomarker that more accurately describes the overall risk of impaired immune responses compared to absolute CD4^+^ T-cell counts and HIV-1 viral loads^27^. A ratio above 1.0 is considered normal but can be influenced by various factors such as age, sex, and ethnicity^27,28^. A lower CD4^+^/CD8^+^ T-cell ratio generally predicts adverse clinical outcomes^28^, whereas ratios above 2.4 have been associated with increased risk of fatal outcomes during acute Covid-19^29^. The observed lower CD4^+^/CD8^+^ T-cell ratios in WLWH (< 1.0) compared with higher ratios in HIV-uninfected women (> 1.5) can be attributed to a greater proportion of CD3^+^ T cells with a CD8^+^ phenotype, which did not normalize despite ART^30^. While the duration of ART could potentially be a confounding factor, it is important to note that information on ART duration was not reported for the participants enrolled in our study.

Reduced numbers of CD4^+^ T cells in PWH have been linked to suboptimal SARS-CoV-2-specific humoral and cell-mediated immune responses^31^. SARS-CoV-2-specific CD4^+^ T cells not only play a critical role in controlling and resolving acute Covid-19^32–34^, but also support long-term immunity by assisting CD8^+^ T cells and B cells^35,36^. Previous studies have linked SARS-CoV-2-specific T-cell responses in Black Africans living with uncontrolled HIV infection to impaired T-cell immunity, characterised by lower T-cell response magnitudes, reduced polyfunctionality, and diminished cross-recognition within CD4^+^ and CD8^+^ T-cell subsets^37,38^. We demonstrated that WLWH on ART exhibited comparable T-cell responses and polyfunctionality profiles compared with HIV-uninfected women, despite having a lower CD4^+^/CD8^+^ T-cell ratio (< 1.0) and increased proportions of CD8^+^ T cells. Our findings corroborate observations from a previous study by our group in similarly healthy African adults on ART who received the ChAdOX1 nCoV-19 (AZD1222) vaccine^39^.This suggests that pregnant and postpartum WLWH on effective ART and who are virally suppressed could experience similarly favourable clinical outcomes as HIV-uninfected women. However, it remains critical to investigate how the interplay between SARS-CoV-2 and HIV co-infection causes other immunological disparities that could compromise the ability of PWH to recover from Covid-19^40^.

Expression of the classic exhaustion marker PD-1 (CD279) on CD4^+^ and CD8^+^ T cells during SARS-CoV-2 infection has been associated with Covid-19 disease progression^41^. Not only do the proportions of PD-1-expressing CD4^+^ and CD8^+^ T cells increase during acute and chronic SARS-CoV-2 infection, but also their relative expression levels of PD-1. PD-1 acts as a down-regulator of immune responses and a suppressor of T-cell inflammatory activity^42^. However, recent evidence suggests that increased PD-1 expression indicates ongoing activation of these cells rather than functional exhaustion^43^. PWH exhibit increased frequencies of CD4^+^ and CD8^+^ T cells expressing PD-1 compared to HIV-uninfected individuals during SARS-CoV-2 infection^31,37,44^. Although we could not differentiate PD-1 expression between different CD4^+^ and CD8^+^ T-cell subsets, we demonstrated similar PD-1 expression levels among women living with and without HIV one month post positive SARS-CoV-2 diagnosis, with similar SARS-CoV-2 viral loads at the time of diagnosis. Furthermore, we showed significantly increased proportions of PD-1-expressing CD8^+^ T cells in WLWH relative to HIV-uninfected women, which we postulate is likely a consequence of long-term chronic HIV-1 infection rather than acute SARS-CoV-2 infection alone^45^. Immune activation persists in PWH despite ART, contributing to a more exhausted immune profile as evidenced by increased proportions of PD-1 expressing CD8^+^ T cells^45–47^.

During pregnancy, there is an upregulation of innate immune responses, as well as a natural shift from Th1 immune responses to Th2 responses^48^. The postpartum period may reflect aspects of pregnancy-associated alterations for an unknown duration, and the return to a pre-pregnant immune state is not well characterized, although it may take up to 12 months^49^. This may explain why we observed similar T-cell responses between pregnant and postpartum women living with and without HIV. Other studies have similarly indicated that the SARS-CoV-2-specific CD4^+^ T-cell response magnitude outnumbers the CD8^+^ T-cell response magnitude^50,51^. However, 15-mer peptide antigens used in these studies may have underrepresented the contribution of SARS-CoV-2-specific CD8^+^ T-cell responses, as they are optimal for HLA class II binding and have been reported to capture on average only 77% of CD8^+^ T-cell responses compared to shorter peptides^52^.

Lastly, we demonstrated robust CD4^+^ and CD8^+^ T-cell responses that cross-react with Omicron to a similar degree compared with WT SARS-CoV-2, irrespective of HIV status. Our results are consistent with other studies investigating potential T-cell escape by Beta, Delta, and Omicron variants^51,53,54^. Despite spike-specific mutations in SARS-CoV-2 variants, which may diminish the cross-reactivity of certain T-cell epitopes, broad T-cell responses develop following SARS-CoV-2 infection in response to immunodominant epitopes located across the viral proteome^55,56^. Therefore, despite spike-specific mutations found in different SARS-CoV-2-variants, T-cell immunity is mostly conserved and peaks between days 14–30 post-infection^37,51,54^.

Our study had some limitations. We only characterized SARS-CoV-2-specific T-cell immunity by focusing on the ability of T-lymphocytes to produce IL-2, IFN-γ, and TNF-α, which are characteristic of a Th1 immune response. Since we included pregnant and postpartum women living with and without HIV, investigating Th2 immune responses would have provided additional information about the immunological landscape^57,58^. Furthermore, we had limited numbers of WLWH at one month post-SARS-CoV-2 infection, which limits the power of our analyses and the interpretation of results. This may explain why we were unable to identify apparent patterns in polyfunctionality profiles between pregnant, postpartum, and non-pregnant women living with HIV. We did not perform viral genome sequencing following confirmed SARS-CoV-2 infection to confirm the identity of the variant. The identity of the SARS-CoV-2 variants was inferred solely by temporal association in relation to the enrolment periods that coincided with variant-specific Covid-19 waves in South Africa. The possibility remains that seasonal human coronaviruses or other SARS-CoV-2 variants could have been responsible for the detected T-cell responses, not just WT SARS-CoV-2, Beta variant, or Delta variant. Lastly, since these women were tested for SARS-CoV-2 infection during routine antenatal care, positive cases were purely incidental detections. We do not know exactly when the infections occurred and how the results might have differed for women with severe Covid-19.

In conclusion, SARS-CoV-2 infection induced comparable T-cell responses and polyfunctionality profiles between pregnant and postpartum women living with and without HIV who were naïve to Covid-19 vaccines. Since T-cell immunity develops in response to immunodominant epitopes located across the viral proteome, our investigation focusing on only two immunodominant structural proteins provides reassurance for HIV-burdened low- and middle-income settings, where a significant proportion of the population were infected before Covid-19 vaccination. Additionally, cross-reactive T cells against Omicron and WT SARS-CoV-2 were elicited to a similar degree in women infected with the ancestral virus, Beta variant, or Delta variant, confirming the overall preservation of T-cell immunity despite variant-specific spike mutations.

## Methods

### Study participants

Women attending routine antenatal care at Chris Hani Baragwanath Academic Hospital (CHBAH) and Rahima Moosa Mother and Child Hospital (RMMCH) in Johannesburg, South Africa, were invited to enrol into a longitudinal cohort study of pregnant women during the antepartum period to investigate the association between SARS-CoV-2 infection and adverse birth outcomes. Participants underwent screening for SARS-CoV-2 infection using nucleic acid amplification test (NAAT), as previously described^4^. Upon incidental detection of a positive SARS-CoV-2 NAAT result, participants were requested to visit the study clinic approximately 1 month later (between 14 and 50 days), at which time venous blood was collected and peripheral blood mononuclear cells (PBMCs) were isolated; this may have occurred during pregnancy or postpartum. Overall, 39 women provided convalescent blood samples: 17 were still pregnant (HIV-uninfected: *n* = 12; WLWH *n* = 5); 17 were postpartum (HIV-uninfected: *n* = 12; WLWH: *n* = 5); and five were not pregnant (WLWH: *n* = 5; control group). Informed consent was obtained from all individual participants included in this study.

Participants were enrolled during three periods: April 1, 2020, to October 31, 2020; November 1, 2020, to April 30, 2021; and May 1, 2021, to November 30, 2021. These recruitment periods coincided with the first, second and third waves of Covid-19 in South Africa, driven by WT SARS-CoV-2, Beta variant (B.1.351), and Delta variant (B.1.617.2), respectively. The identity of the causative SARS-CoV-2 variant was determined solely by temporal association and was not confirmed by viral genome sequencing. The current study was approved by the University of the Witwatersrand Human Research Ethics Committee (HREC: M220285). Clinical data were collected and managed electronically using REDCap^®^ (v.13.5.4; Vanderbilt University, Nashville, Tennessee, US) hosted by the University of the Witwatersrand^59,60^.

### Peripheral blood mononuclear cell isolation and storage

PBMCs were isolated from heparinized blood samples using density gradient centrifugation with Ficoll-Paque™ PLUS (Cytiva, Uppsala, Sweden) and cryopreserved in freezing media composed of 90% heat-inactivated fetal bovine serum (FBS; Gibco™, Life Technologies, Paisley, UK) and 10% dimethyl sulfoxide (DMSO; Sigma-Aldrich, St Louis, MO, USA).

### SARS-CoV-2 peptide antigens

Commercially available lyophilized PepTivator^®^ SARS-CoV-2 peptide pools (Miltenyi Biotec, Bergisch Gladbach, Germany), consisting of 15-mer sequences with 11 amino acid (aa) overlap, were used to stimulate SARS-CoV-2-specific CD4^+^ and CD8^+^ T cells in vitro. Peptide pools Prot_S1 (Cat# 130-127-048) and Prot_S (Cat# 130-126-701) were combined to represent the near full-length spike (FLS) glycoprotein of WT SARS-CoV-2, as previously described^51^. Prot_S1 covers the N-terminal S1 domain (aa 1–692), whereas Prot_S covers the C-terminal S2 domain spanning aa 683–707, 741–770, 785–802, and 885–1273 (GenBank MN908947.3, Protein QHD43416.1). Prot_N (Cat# 130-126-699) represented the complete nucleocapsid (N) protein of WT SARS-CoV-2 (GenBank MN908947.3, Protein QHD43423.2). For the Omicron variant (B.1.1.529), Prot_S B.1.1.529/BA.5 (Cat# 130-132-051) was used, consisting of 76 peptides covering 34 selectively mutated S-protein regions unique to lineages BA.4 and BA.5, including mutations: T19I, ΔL24/P25/P26, A27S, ΔH69/V70, G142D, V213G, G339D, S371F, S373P, S375F, T376A, D405N, R408S, K417N, N440K, L452R, S477N, T478K, E484A, F486V, Q498R, N501Y, Y505H, D614G, H655Y, N679K, P681H, N764K, D796Y, Q954H, N969K. A corresponding reference pool (Cat# 130-132-050), containing 76 homologous peptides of WT SARS-CoV-2, was included as a control for the variant pool. The lyophilized peptide pools were reconstituted using distilled water.

### Cell stimulation and flow cytometry staining

Cryopreserved PBMCs were thawed as previously described^61^ and rested for 4 h in R10 medium (RMPI-1640 medium (Sigma-Aldrich) supplemented with 10% heat-inactivated FBS (Gibco™), 1 mM penicillin-streptomycin (Gibco™), and 2 mM l-glutamine (Gibco™)). A total of 1.5–2.0 × 10^6^ PBMCs were seeded per reaction well in a 96-well U-bottom plate (Thermo Scientific™, Waltham, Massachusetts, US) and stimulated with SARS-CoV-2-specific peptide pools (2 µg/mL; Miltenyi Biotec) for 16 h at 37 °C and 5% CO_2_. All stimulations were performed in the presence of brefeldin A (10 µg/mL, BFA; Sigma-Aldrich) and co-stimulatory antibodies against CD28 (1 µg/mL, clone CD28.2) and CD49d (1 µg/mL, clone 9F10) (BD Pharmingen™, BD Biosciences, San Jose, California, US). An unstimulated control, containing BFA and co-stimulatory antibodies, was included per participant. Additionally, a plate control consisted of PBMCs from a Covid-19 vaccinated donor stimulated with phytohemagglutinin-L (1 µg/mL, PHA; Sigma-Aldrich). After stimulation, cells were washed and stained with LIVE/DEAD™ Fixable Yellow Stain (1:1000; Invitrogen™, Carlsbad, California, US). Subsequently, cells were surface stained with the following antibodies: CD4 BV785 (1:100, clone OKT4; BioLegend, San Diego, California, US), CD8a BV510 (1:100, clone RPA-T8; BioLegend), and CD279 (PD-1) BB515 (1:100, clone EH12.1; BD Horizon™, BD Biosciences). Cells were then fixed and permeabilized using Fixation/Permeabilization solution (BD Biosciences) before staining with CD3 BV650 (1:100, clone OKT3; BioLegend) and intracellular antibodies: IFN-γ BV711 (1:100, clone 4 S.B3; BioLegend), TNF-α PE-Cy7 (1:100, clone MAb11; BioLegend), and IL-2 PE-Dazzle-594 (1:100, clone MQ1-17H12; BioLegend). Antibody staining was performed at 22–25 °C for 30 min in the dark. Finally, cells were washed with BD Perm/Wash™ Buffer (1X; BD Biosciences) and resuspended in 300 µL BD FACSFlow™ solution (BD Biosciences). Stained cells were acquired using a 4-laser BD LSRFortessa™ X-20 flow cytometer (BD Biosciences) and analysed with FlowJo™ software (v.10.8.1; FlowJo LLC., Ashland, Oregon, US). Optimal photomultiplier tube voltages for each fluorescent target were established before data acquisition using BD^®^ CS&T beads (BD Biosciences) and maintained throughout experiments using Sphero™ rainbow fluorescent particles (3.0–3.4 μm, mid-range FL1 fluorescence; BD Biosciences). All data are presented after subtracting background signals from unstimulated controls. A representative gating strategy is provided in Supplementary Fig. S1.

### Statistical analysis

Demographic and clinical characteristics were summarised using medians with interquartile ranges (IQRs) for quantitative variables (age, cycle threshold (CT) values, days between PBMC collection and positive SARS-CoV-2 NAATs), and counts with percentages (proportions) for categorical variables (Covid-19 wave periods and ART regimens). Positive T-cell responses were defined as values ≥ 0.002%. Non-responders with T-cell responses equal to 0% were graphically represented with a value of 0.0015%. The Mann–Whitney U-test and Kruskal–Wallis H-test were used to compare responses between two and three unpaired groups, respectively. The Wilcoxon singed-rank test was used for comparing responses between two paired groups. Spearman’s rank correlation coefficient (two-sided) was used to describe the linear relationship between two continuous variables. Statistical analysis and graphical representation were performed using STATA (v.13.1; StataCorp LLC, College Station, Texas, US) and GraphPad Prism (v.10.0.2; GraphPad Software Inc., San Diego, California, US), respectively. *p*-values < 0.05 were considered statistically significant.

### Ethics approval

This is a sub-study of a main study, entitled “Sentinel, hospital-based surveillance for the investigation of SARS-Coronavirus-2 and other respiratory pathogens” which was performed in line with the principles of the Declaration of Helsinki. Approval was obtained from the Human Research Ethics Committee (HREC) of the University of the Witwatersrand (Approval date: April 3, 2020; Reference number: 200313). The current study was also approved by the University of the Witwatersrand’s HREC (Approval date: March 9, 2022; Reference number: M220285).

### Consent to participate

 Informed consent was obtained from all individual participants included in this study.

### Supplementary Information

Supplementary Figures.

## Data Availability

The datasets used and/or analysed during the current study will be made available upon reasonable request directed to the corresponding author.
