# Supplementary material for: T-cell responses to ancestral SARS-CoV-2 and Omicron variant among unvaccinated pregnant and postpartum women living with and without HIV in South Africa
Source: Sci Rep. 2024 Sep 2;14:20348. doi: 10.1038/s41598-024-70725-8 (PMC11369237; doi:10.1038/s41598-024-70725-8)
Supplement: Supplementary file 1 — Supplementary Figures. [file 41598_2024_70725_MOESM1_ESM.pdf]

T-cell responses to ancestral SARS-CoV-2 and Omicron variant among unvaccinated pregnant and postpartum women living with and without HIV in South Africa

William C. McMahon[1,2], Gaurav Kwatra[1,3,4,*], Alane Izu[1,5], Stephanie A. Jones[1], Nkululeko J. Mbele[1], Nwabisa Jafta[1], Rushil Lala[1], Sharon Shalekoff[6,7], Caroline T. Tiemessen[6,7], Shabir A. Madhi[1,5¶], Marta C. Nunes[1,2,8¶]

SUPPLEMENTARY INFORMATION

Supplementary figure legends

Fig. S1. Flow cytometry gating strategy and representative staining profiles.
The flow cytometry gating strategy and representative staining profiles of two plate controls (unstimulated and PHA-stimulated) using cells from a Covid-19 vaccinated donor are shown. Firstly, a time gate was applied to ensure analysis of data with a consistent flow rate. Doublets were excluded by gating FSC-A against FSC-H, followed by gating on lymphocytes using their FSC-A and SSC-A properties. Approximately 100,000 live $CD3^+$ cells were acquired for each reaction. $CD4^+$ and $CD8^+$ T cells were gated and assessed for surface expression of PD-1 (histogram plots), as well as for intracellular expression of IFN-γ, TNF-α, and IL-2 (pseudocolour plots). Percentages indicating responses are presented in each plot.

Fig. S2. Overall median comparisons of SARS-CoV-2-specific $CD4^+$ and $CD8^+$ T-cell responses in women living with and without HIV.
Direct median comparisons between $CD4^+$ and $CD8^+$ T-cell polyfunctionality profiles in response to the (a) FLS and (b) N proteins on WT SARS-CoV-2 among women living with and without HIV. Each point represents the median T-cell response of positive responders for each group of cytokines produced. $CD4^+$ T-cell responses are indicated in green and blue. CD8+ T-cell responses are indicated in red and orange. The Wilcoxon signed-rank test was used for comparing paired $CD4^+$ and $CD8^+$ T-cell responses. Significant P-values are indicated in bold ($p<0.05$). Denotations: FLS, full-length spike glycoprotein; IFN-γ, interferon gamma; Il-2, interleukin 2; N, nucleocapsid protein; TNF-α, tumor necrosis factor alpha; WLWH, women living with HIV; WT, wild-type (ancestral) SARS-CoV-2.

Fig. S3. Maternal T-cell responses to the FLS and N proteins of WT SARS-CoV-2.
The proportion of T-cells producing IL-2, IFN-γ, or TNF-α in response to the FLS and N proteins of WT SARS-CoV-2, stratified by maternal (pregnant: n=17; postpartum: n=16; not pregnant: n=5) and HIV status (HIV-uninfected: n=23; WLWH: n=15). SARS-CoV-2-specific $CD4^+$ T-cell responses to (a) the FLS and (b) N proteins. SARS-CoV-2-specific $CD8^+$ T-cell responses to (c) the FLS and (d) N proteins. Each participant is represented by an individual point, and medians in each group are indicated by horizontal lines. Statistical analysis was performed using the Mann-Whitney U-test for T-cell response comparisons between unpaired (HIV-uninfected vs. WLWH) responders. Significant P-values are indicated in bold ($p<0.05$). Denotations: FLS, full-length spike glycoprotein; HIV-, HIV-uninfected women N, nucleocapsid protein; WLWH, women living with HIV; WT, wild-type (ancestral) SARS-CoV-2.

Fig. S4. Maternal polyfunctionality of $CD4^+$ T-cells to the FLS and N proteins of WT SARS-CoV-2 in HIV-uninfected women.
(a) Comparisons of the polyfunctional profiles of $CD4^+$ T cells to the FLS and N proteins of WT SARS-CoV-2 in HIV-uninfected women, stratified by maternal status. Each participant is represented by an individual point, and medians in each group are indicated by horizontal lines. (b) Each response pattern is colour-coded, and data are summarized in pie charts. The Mann-Whitney U-test was used for comparing unpaired $CD4^+$ T-cell responses of positive responders between pregnant and postpartum

women. Only significant response differences are indicated ($p<0.05$). (c) Correlations between FLS- and N-specific $CD4^+$ T-cell responses according to IL-2, IFN-γ, and TNF-α production. Spearman's rank correlation coefficient (two-sided) was used to determine the *P* and *r* (correlation coefficient) values. Linear regression lines are indicated in red, together with 95% confidence interval lines. Denotations: FLS, full-length spike glycoprotein; HIV-, HIV-uninfected women; IFN-γ, interferon gamma; Il-2, interleukin 2; N, nucleocapsid protein; TNF-α, tumor necrosis factor alpha; WT, wild-type (ancestral) SARS-CoV-2.

Fig. S5. Maternal polyfunctionality of $CD4^+$ T-cells to the FLS and N proteins of WT SARS-CoV-2 in women living with HIV.
(a) Comparisons of the polyfunctional profiles of $CD4^+$ T cells to the FLS and N proteins of WT SARS-CoV-2 in WLWH, stratified by maternal status. Each participant is represented by an individual point, and medians in each group are indicated by horizontal lines. (b) Each response pattern is colour-coded, and data are summarized in pie charts. The Mann-Whitney U-test was used for comparing unpaired $CD4^+$ T-cell responses of positive responders between pregnant, postpartum, and non-pregnant women. Only significant response differences are indicated ($p<0.05$). (c) Correlations between FLS- and N-specific $CD4^+$ T-cell responses according to IL-2, IFN-γ, and TNF-α production. Spearman's rank correlation coefficient (two-sided) was used to determine the *P* and *r* (correlation coefficient) values. Linear regression lines are indicated in red, together with 95% confidence interval lines. Denotations: FLS, full-length spike glycoprotein; IFN-γ, interferon gamma; IL-2, interleukin 2; N, nucleocapsid protein; TNF-α, tumor necrosis factor alpha; WLWH, women living with HIV; WT, wild-type (ancestral) SARS-CoV-2.

Fig. S6. Maternal polyfunctionality of $CD8^+$ T-cells to the FLS and N proteins of WT SARS-CoV-2 in HIV-uninfected women.
(a) Comparisons of the polyfunctional profiles of $CD8^+$ T cells to the FLS and N proteins of WT SARS-CoV-2 in HIV-uninfected women, stratified by maternal status. Each participant is represented by an individual point, and medians in each group are indicated by horizontal lines. (b) Each response pattern is colour-coded, and data summarized in pie charts. The Mann-Whitney U-test was used for comparing unpaired $CD8^+$ T-cell responses of positive responders between pregnant and postpartum women. Only significant response differences are indicated ($p<0.05$). (c) Correlations between FLS- and N-specific $CD8^+$ T-cell responses according to IL-2, IFN-γ, and TNF-α production. Spearman's rank correlation coefficient (two-sided) was used to determine the *P* and *r* (correlation coefficient) values. Linear regression lines are indicated in red, together with 95% confidence interval lines. Denotations: FLS, full-length spike glycoprotein; HIV-, HIV-uninfected women; IFN-γ, interferon gamma; IL-2, interleukin 2; N, nucleocapsid protein; TNF-α, tumor necrosis factor alpha; WT, wild-type (ancestral) SARS-CoV-2.

Fig. S7. Maternal polyfunctionality of CD8+ T-cells to the FLS and N proteins of WT SARS-CoV-2 in women living with HIV.
(a) Comparisons of the polyfunctional profiles of $CD8^+$ T cells to the FLS and N proteins of WT SARS-CoV-2 in WLWH, stratified by maternal status. Each participant is represented by an individual point, and medians in each group are indicated by horizontal lines. (b) Each response pattern is colour-coded, and data summarized in pie charts. The Mann-Whitney U-test was used for comparing unpaired $CD8^+$ T-cell responses of positive responders between pregnant, postpartum, and non-pregnant women. Only significant response differences are indicated ($p<0.05$). (c) Correlations between FLS- and N-specific $CD4^+$ T-cell responses according to IL-2, IFN-γ, and TNF-α production. Spearman's rank correlation coefficient (two-sided) was used to determine the *P* and *r* (correlation coefficient) values. Linear regression lines are indicated in red, together with 95% confidence interval lines. Denotations: FLS, full-length spike glycoprotein; IFN-γ, interferon gamma; IL-2, interleukin 2; N, nucleocapsid protein; TNF-α, tumor necrosis factor alpha; WLWH, women living with HIV; WT, wild-type (ancestral) SARS-CoV-2.

Fig. S8, Cytokine median fluorescence intensities of CD4$^+$ and CD8$^+$ T-cells to the FLS and N proteins of WT SARS-CoV-2 in women living with and without HIV.

Comparisons of individual cytokine median fluorescence intensities (MFIs) of (a) CD4$^+$ and (b) CD8$^+$ T cells in response to the FLS- and N-proteins of WT SARS-CoV-2, stratified by HIV status. Each participant is represented by an individual point, and medians in each group are indicated by horizontal lines. The Mann-Whitney U-test was used for comparing unpaired MFIs between women living with and without HIV. The Wilcoxon singed-rank test and Friedman test were used for comparing MFIs between two and three paired groups, respectively. Denotations: FLS, full-length spike glycoprotein; IFN-γ, interferon gamma; IL-2, interleukin 2; MFI, median fluorescence intensity; N, nucleocapsid protein; TNF-α, tumor necrosis factor alpha; WLWH, women living with HIV; WT, wild-type (ancestral) SARS-CoV-2.

Fig. S9, Cytokine median fluorescence intensities of CD4$^+$ and CD8$^+$ T-cells to WT SARS-CoV-2 and the Omicron variant in women living with and without HIV.

Comparisons of individual cytokine median fluorescence intensities (MFIs) of (a) CD4$^+$ and (b) CD8$^+$ T cells in response to spike proteins of WT SARS-CoV-2 and the Omicron variant, stratified by HIV status. Each participant is represented by an individual point, and medians in each group are indicated by horizontal lines. The Mann-Whitney U-test was used for comparing unpaired MFIs between women living with and without HIV. The Wilcoxon singed-rank test and Friedman test were used for comparing MFIs between two and three paired groups, respectively. Denotations: FLS, full-length spike glycoprotein; IFN-γ, interferon gamma; IL-2, interleukin 2; MFI, median fluorescence intensity; N, nucleocapsid protein; TNF-α, tumor necrosis factor alpha; WLWH, women living with HIV; WT, wild-type (ancestral) SARS-CoV-2.

Supplementary figures

Fig. S1

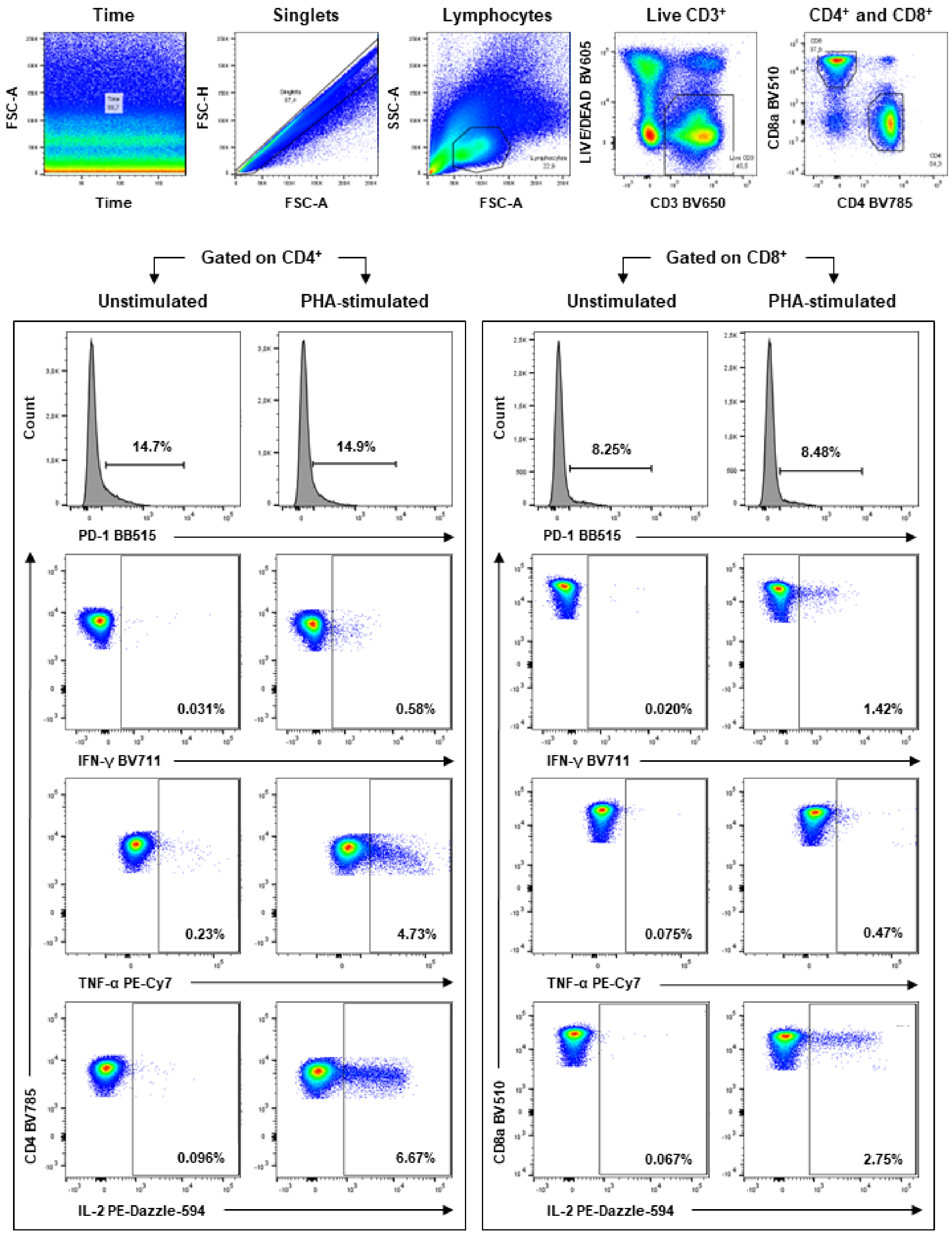

Fig. S2

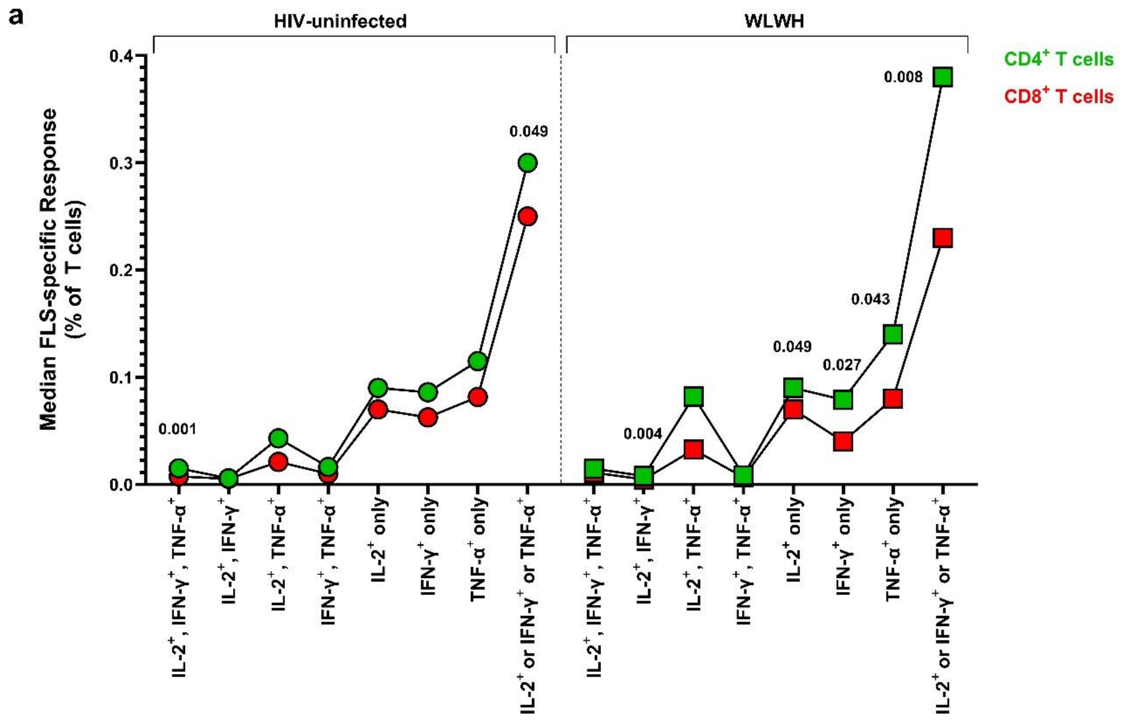
a
HIV-uninfected
WLWH
CD4+ T cells
CD8+ T cells
Median FLS-specific Response (% of T cells)
0.4
0.3
0.2
0.1
0.0
0.001
0.049
0.004
0.049
0.027
0.043
0.008
IL-2+, IFN-γ+, TNF-α+
IL-2+, IFN-γ+
IL-2+, TNF-α+
IFN-γ+, TNF-α+
IL-2+ only
IFN-γ+ only
TNF-α+ only
IL-2+ or IFN-γ+ or TNF-α+

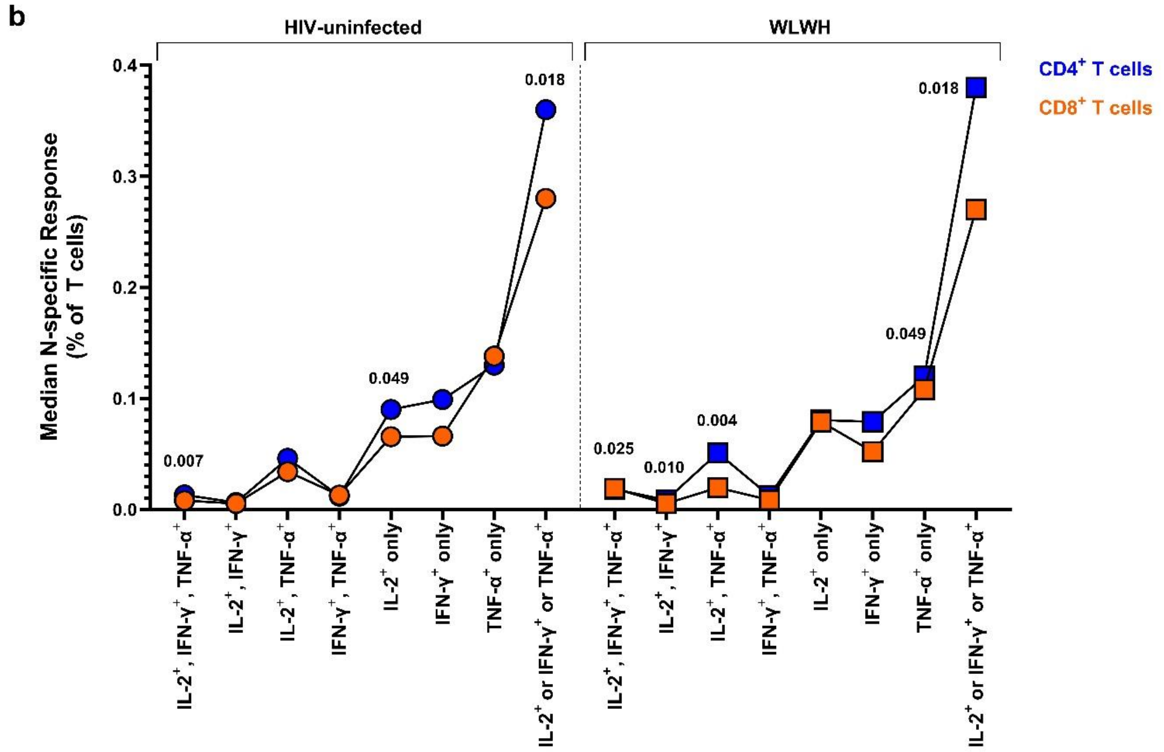
b
HIV-uninfected
WLWH
CD4+ T cells
CD8+ T cells
Median N-specific Response (% of T cells)
0.4
0.3
0.2
0.1
0.0
0.007
0.049
0.018
0.025
0.010
0.004
0.049
0.018
IL-2+, IFN-γ+, TNF-α+
IL-2+, IFN-γ+
IL-2+, TNF-α+
IFN-γ+, TNF-α+
IL-2+ only
IFN-γ+ only
TNF-α+ only
IL-2+ or IFN-γ+ or TNF-α+

Fig. S3

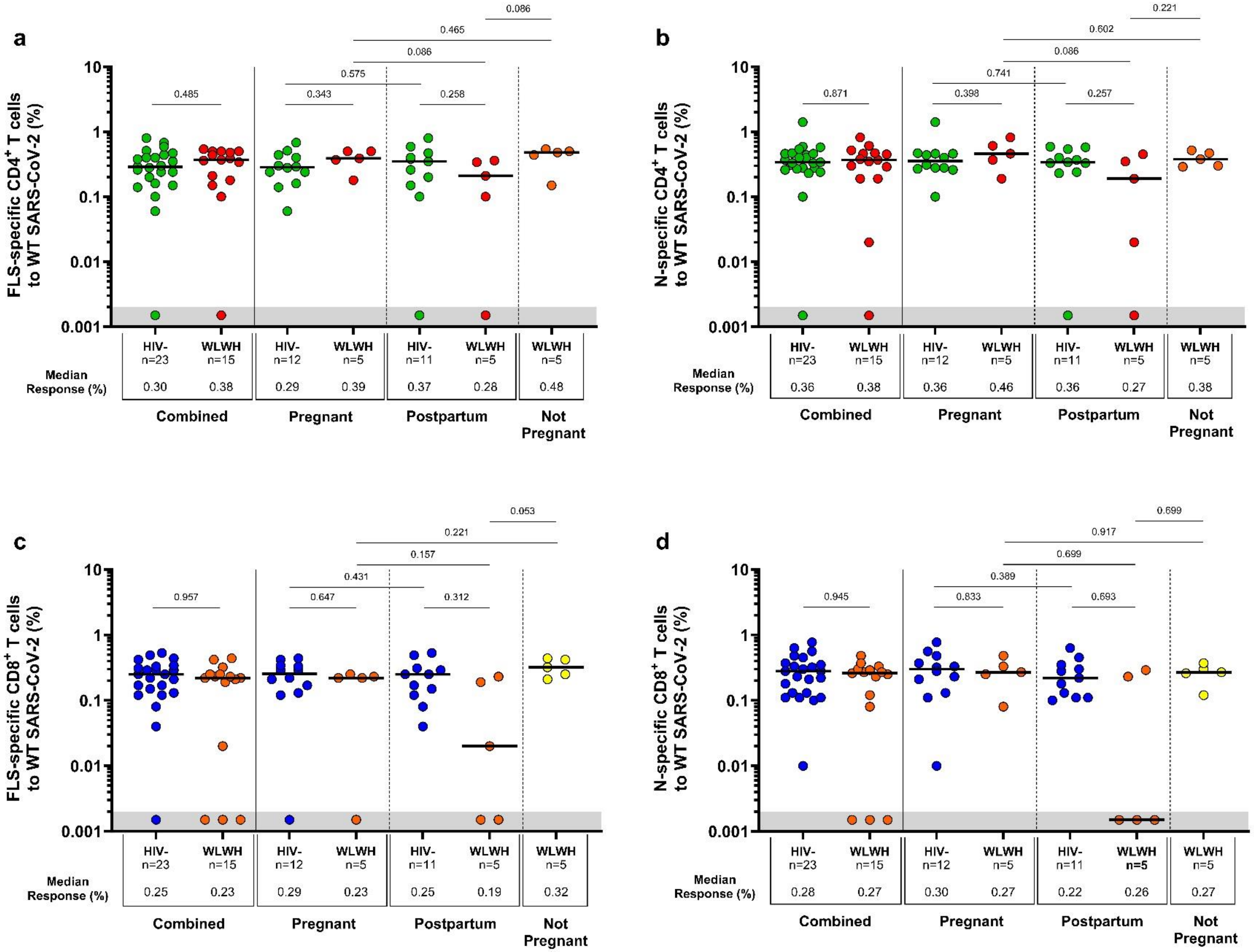

a: Median response (%): Combined HIV- 0.30, WLWH 0.38; Pregnant HIV- 0.29, WLWH 0.39; Postpartum HIV- 0.37, WLWH 0.28; Not Pregnant WLWH 0.48. P values: 0.485, 0.343, 0.575, 0.258, 0.086, 0.465, 0.086.

b: Median response (%): Combined HIV- 0.36, WLWH 0.38; Pregnant HIV- 0.36, WLWH 0.46; Postpartum HIV- 0.36, WLWH 0.27; Not Pregnant WLWH 0.38. P values: 0.871, 0.398, 0.741, 0.257, 0.086, 0.602, 0.221.

c: Median response (%): Combined HIV- 0.25, WLWH 0.23; Pregnant HIV- 0.29, WLWH 0.23; Postpartum HIV- 0.25, WLWH 0.19; Not Pregnant WLWH 0.32. P values: 0.957, 0.647, 0.431, 0.312, 0.157, 0.221, 0.053.

d: Median response (%): Combined HIV- 0.28, WLWH 0.27; Pregnant HIV- 0.30, WLWH 0.27; Postpartum HIV- 0.22, WLWH 0.26; Not Pregnant WLWH 0.27. P values: 0.945, 0.833, 0.389, 0.693, 0.699, 0.917, 0.699.

Fig. S4

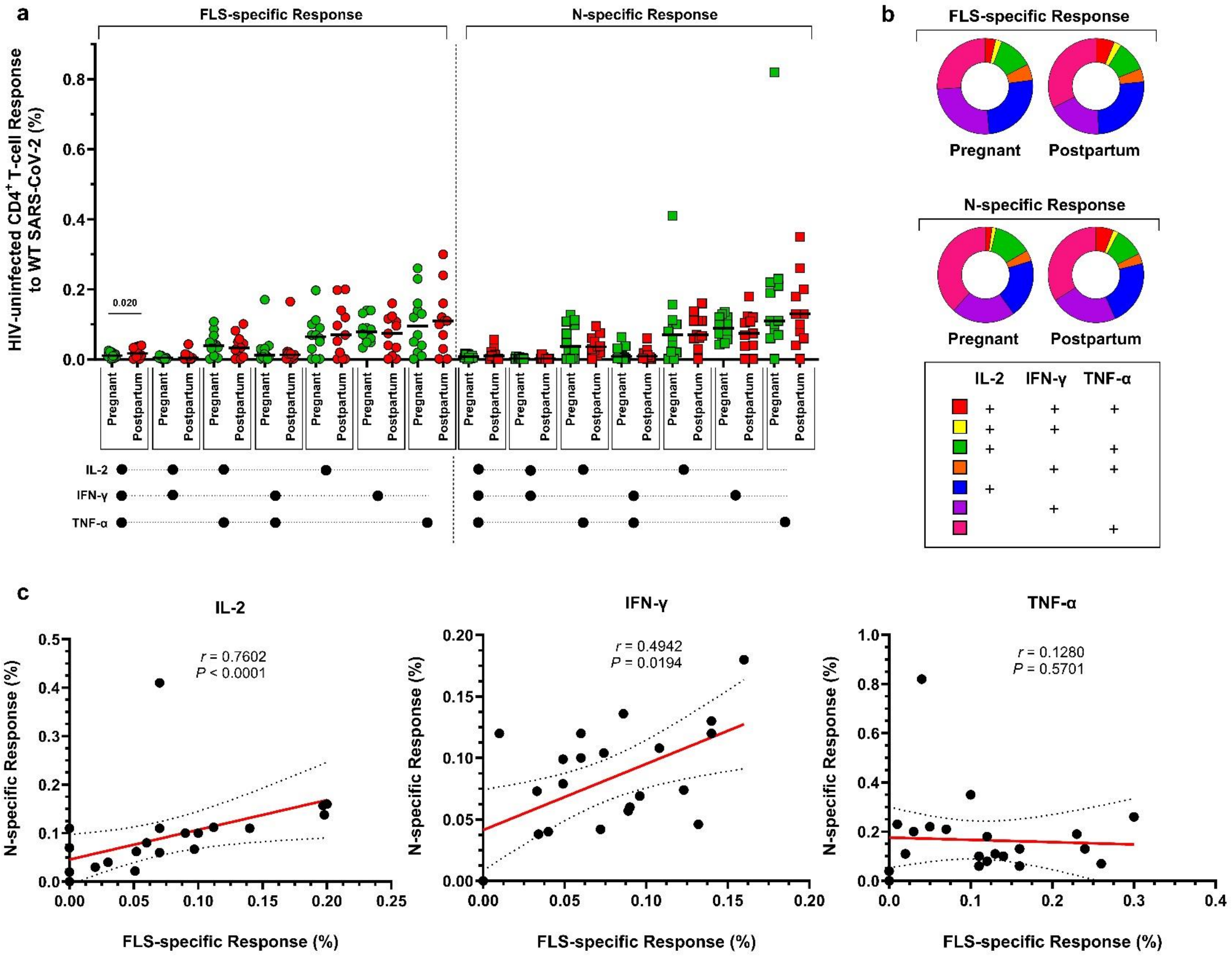
a
FLS-specific Response
N-specific Response
HIV-uninfected CD4+ T-cell Response to WT SARS-CoV-2 (%)
0.020
Pregnant
Postpartum
IL-2
IFN-γ
TNF-α
b
FLS-specific Response
Pregnant
Postpartum
N-specific Response
Pregnant
Postpartum
IL-2 IFN-γ TNF-α
c
IL-2
r = 0.7602
P < 0.0001
N-specific Response (%)
FLS-specific Response (%)
IFN-γ
r = 0.4942
P = 0.0194
TNF-α
r = 0.1280
P = 0.5701

Fig. S5

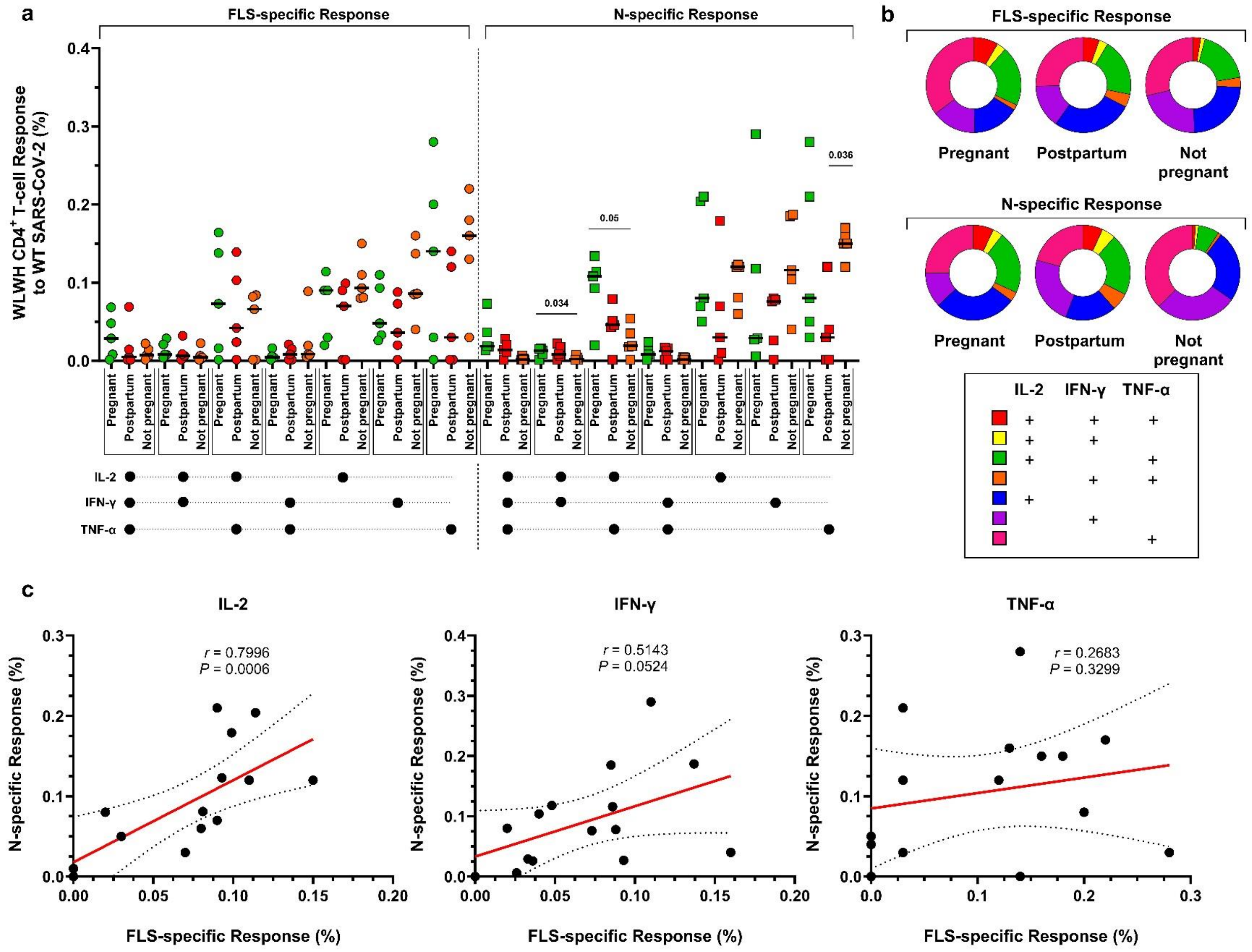
a
FLS-specific Response
N-specific Response
WLWH CD4+ T-cell Response to WT SARS-CoV-2 (%)
0.034
0.05
0.036
Pregnant
Postpartum
Not pregnant
IL-2
IFN-γ
TNF-α
b
FLS-specific Response
Pregnant
Postpartum
Not pregnant
N-specific Response
IL-2
IFN-γ
TNF-α
c
IL-2
r = 0.7996
P = 0.0006
IFN-γ
r = 0.5143
P = 0.0524
TNF-α
r = 0.2683
P = 0.3299
N-specific Response (%)
FLS-specific Response (%)

Fig. S6

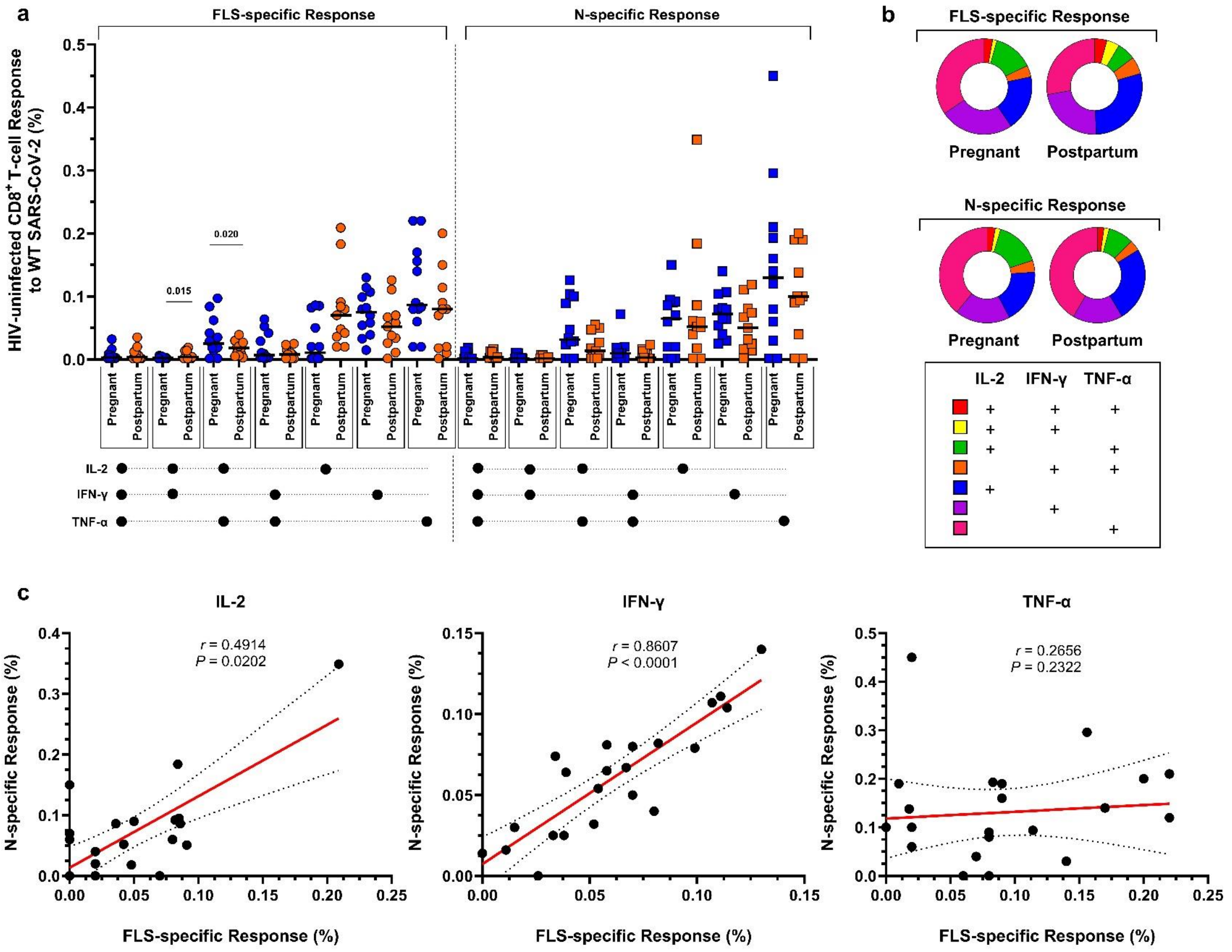
a
FLS-specific Response
N-specific Response
HIV-uninfected CD8+ T-cell Response to WT SARS-CoV-2 (%)
0.015
0.020
Pregnant
Postpartum
IL-2
IFN-γ
TNF-α
b
FLS-specific Response
Pregnant
Postpartum
N-specific Response
Pregnant
Postpartum
IL-2 IFN-γ TNF-α
c
IL-2
r = 0.4914
P = 0.0202
IFN-γ
r = 0.8607
P < 0.0001
TNF-α
r = 0.2656
P = 0.2322
N-specific Response (%)
FLS-specific Response (%)

Fig. S7

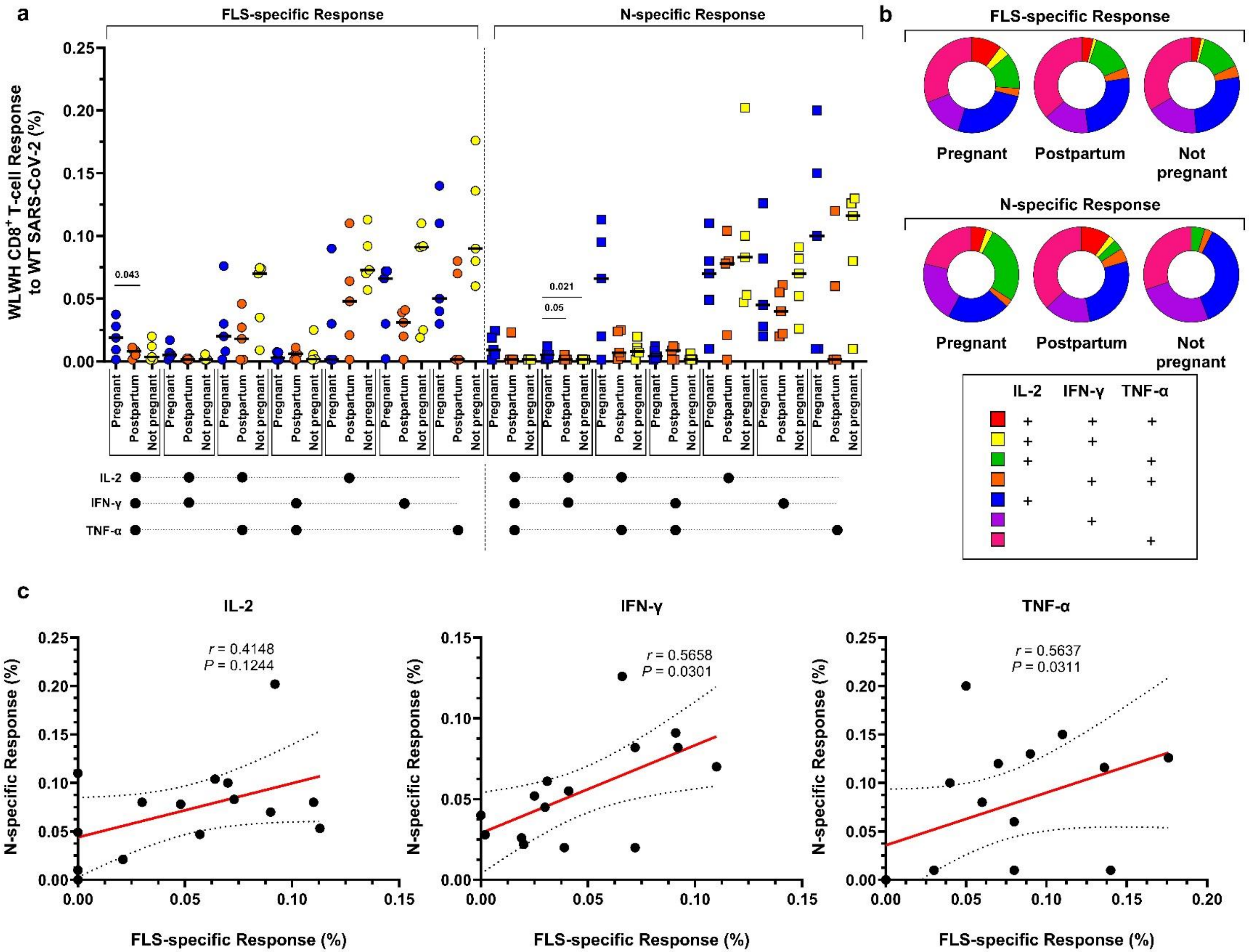
a
FLS-specific Response
N-specific Response
WLWH CD8+ T-cell Response to WT SARS-CoV-2 (%)
0.043
0.021
0.05
Pregnant
Postpartum
Not pregnant
IL-2
IFN-γ
TNF-α
b
FLS-specific Response
Pregnant
Postpartum
Not pregnant
N-specific Response
IL-2
IFN-γ
TNF-α
c
IL-2
r = 0.4148
P = 0.1244
IFN-γ
r = 0.5658
P = 0.0301
TNF-α
r = 0.5637
P = 0.0311
N-specific Response (%)
FLS-specific Response (%)

Fig. S8

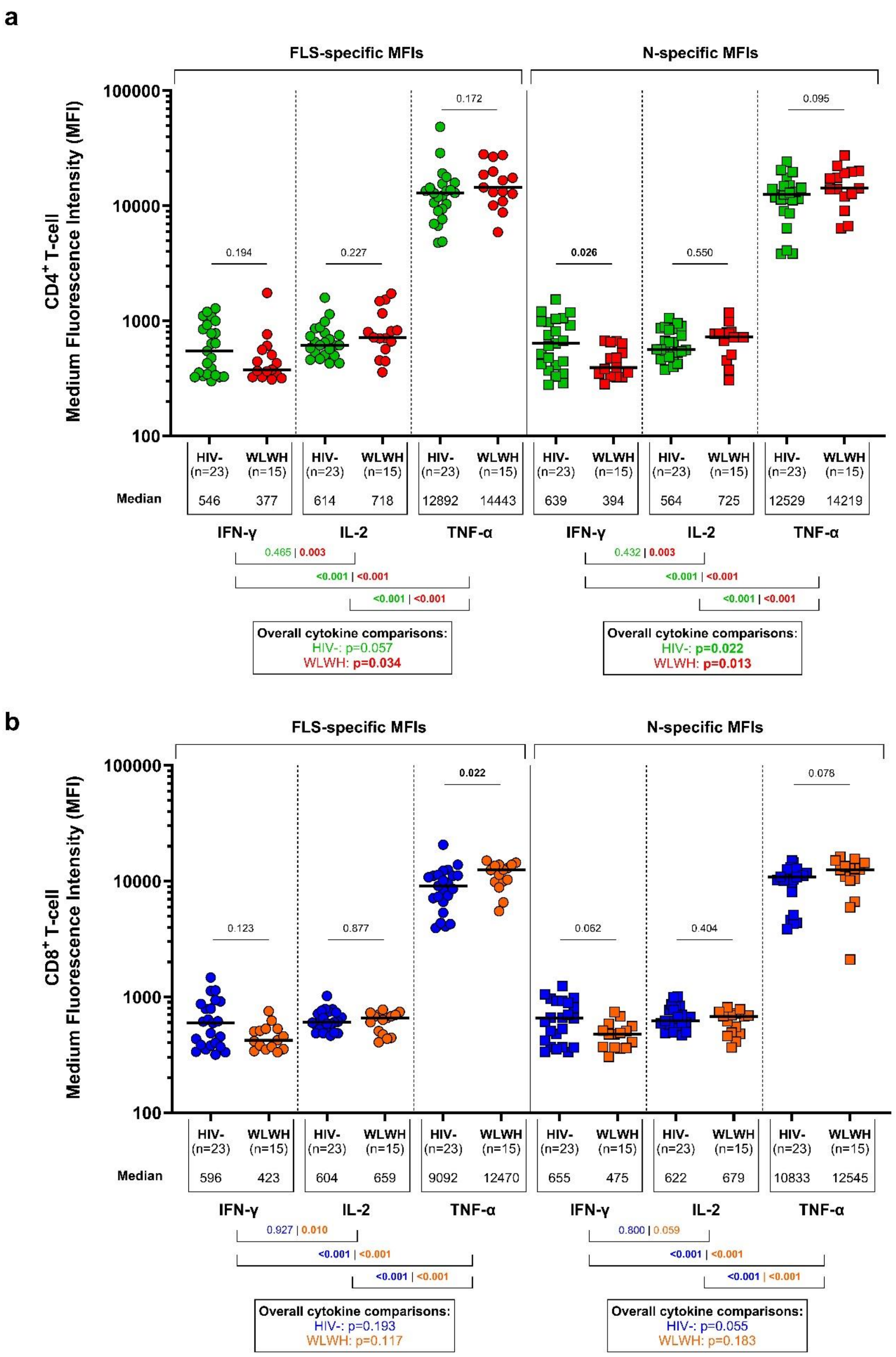
a
FLS-specific MFIs
N-specific MFIs
CD4+ T-cell Medium Fluorescence Intensity (MFI)
100000
10000
1000
100
0.194
0.227
0.172
0.026
0.550
0.095
HIV- (n=23)
WLWH (n=15)
Median
546 377 614 718 12892 14443 639 394 564 725 12529 14219
IFN-γ
IL-2
TNF-α
0.465 | 0.003
<0.001 | <0.001
<0.001 | <0.001
0.432 | 0.003
<0.001 | <0.001
<0.001 | <0.001
Overall cytokine comparisons:
HIV-: p=0.057
WLWH: p=0.034
Overall cytokine comparisons:
HIV-: p=0.022
WLWH: p=0.013
b
FLS-specific MFIs
N-specific MFIs
CD8+ T-cell Medium Fluorescence Intensity (MFI)
100000
10000
1000
100
0.123
0.877
0.022
0.062
0.404
0.078
HIV- (n=23)
WLWH (n=15)
Median
596 423 604 659 9092 12470 655 475 622 679 10833 12545
IFN-γ
IL-2
TNF-α
0.927 | 0.010
<0.001 | <0.001
<0.001 | <0.001
0.800 | 0.059
<0.001 | <0.001
<0.001 | <0.001
Overall cytokine comparisons:
HIV-: p=0.193
WLWH: p=0.117
Overall cytokine comparisons:
HIV-: p=0.055
WLWH: p=0.183

Fig. S9

a

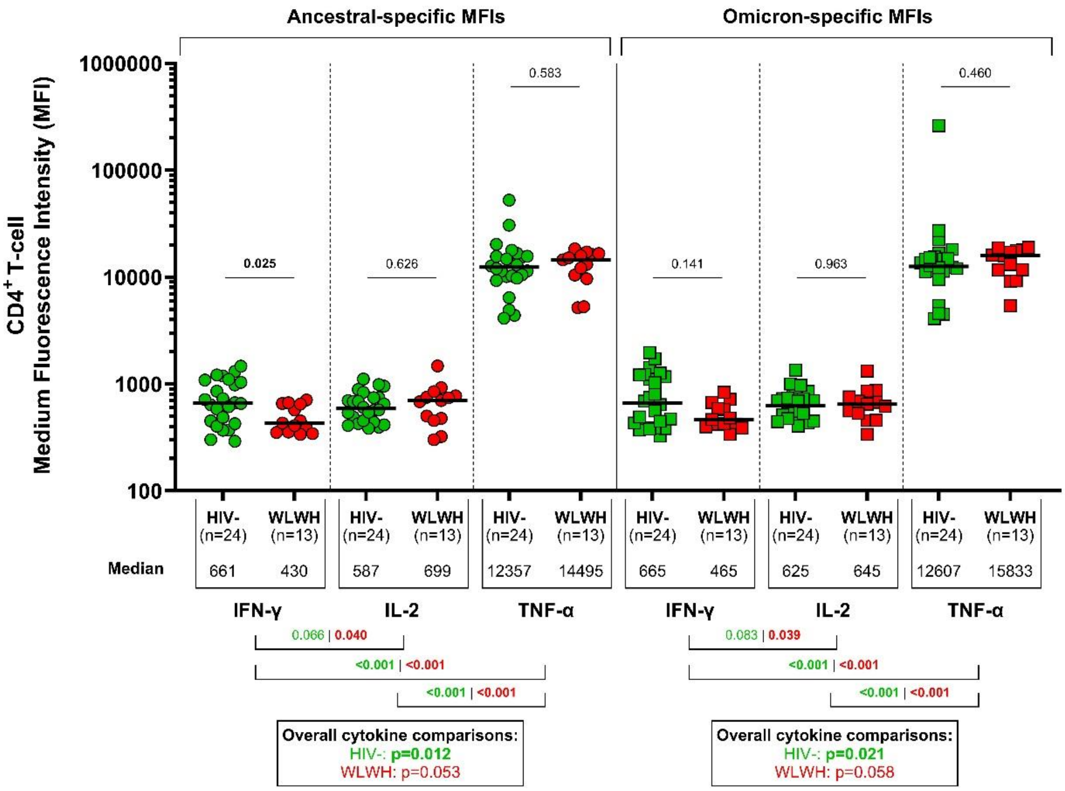
Ancestral-specific MFIs
Omicron-specific MFIs
CD4+ T-cell
Medium Fluorescence Intensity (MFI)
1000000
100000
10000
1000
100
0.025
0.626
0.583
0.141
0.963
0.460
HIV- (n=24)
WLWH (n=13)
Median
661 430 587 699 12357 14495 665 465 625 645 12607 15833
IFN-γ
IL-2
TNF-α
0.066 | 0.040
<0.001 | <0.001
<0.001 | <0.001
0.083 | 0.039
<0.001 | <0.001
<0.001 | <0.001
Overall cytokine comparisons:
HIV-: p=0.012
WLWH: p=0.053
Overall cytokine comparisons:
HIV-: p=0.021
WLWH: p=0.058

b

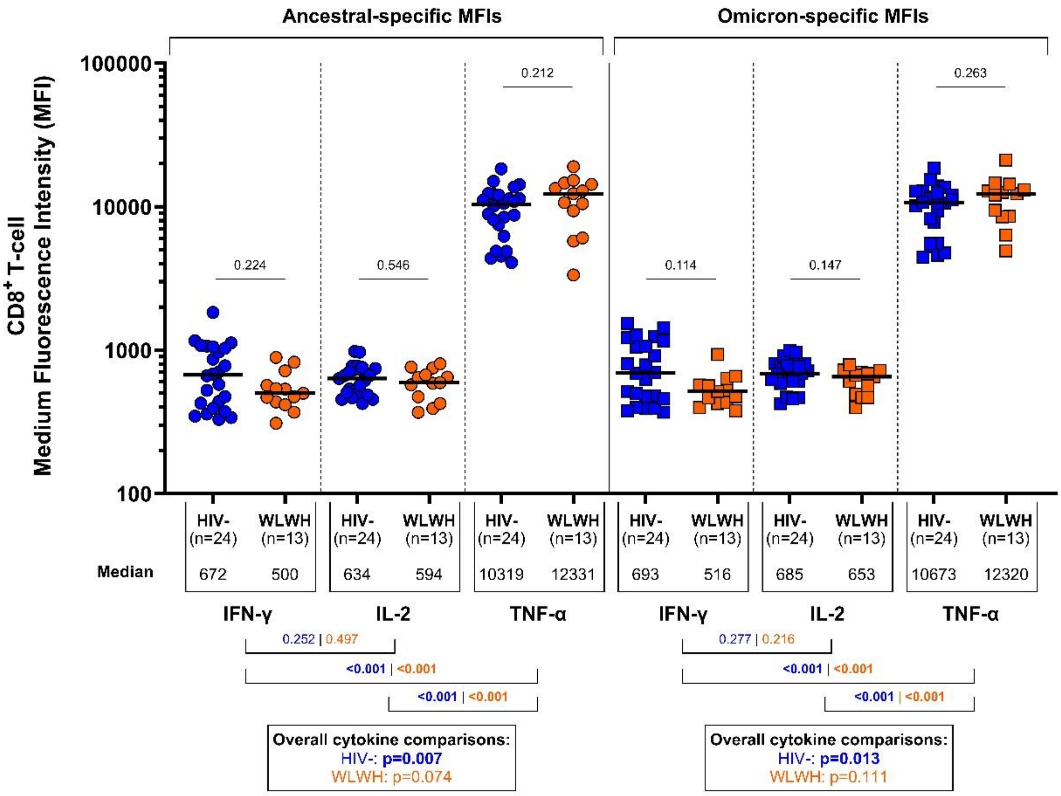
Ancestral-specific MFIs
Omicron-specific MFIs
CD8+ T-cell
Medium Fluorescence Intensity (MFI)
100000
10000
1000
100
0.224
0.546
0.212
0.114
0.147
0.263
HIV- (n=24)
WLWH (n=13)
Median
672 500 634 594 10319 12331 693 516 685 653 10673 12320
IFN-γ
IL-2
TNF-α
0.252 | 0.497
<0.001 | <0.001
<0.001 | <0.001
0.277 | 0.216
<0.001 | <0.001
<0.001 | <0.001
Overall cytokine comparisons:
HIV-: p=0.007
WLWH: p=0.074
Overall cytokine comparisons:
HIV-: p=0.013
WLWH: p=0.111
